# Supplementary material for: Preclinical Study Using ABT263 to Increase Enzalutamide Sensitivity to Suppress Prostate Cancer Progression Via Targeting BCL2/ROS/USP26 Axis Through Altering ARv7 Protein Degradation
Source: Cancers (Basel). 2020 Mar 30;12(4):831. doi: 10.3390/cancers12040831 (PMC7226306; doi:10.3390/cancers12040831)
Supplement: Supplementary file 1 [file cancers-12-00831-s001.zip › cancers-732702 supplementary/Raw data for western blot.pdf]

Figure 1C

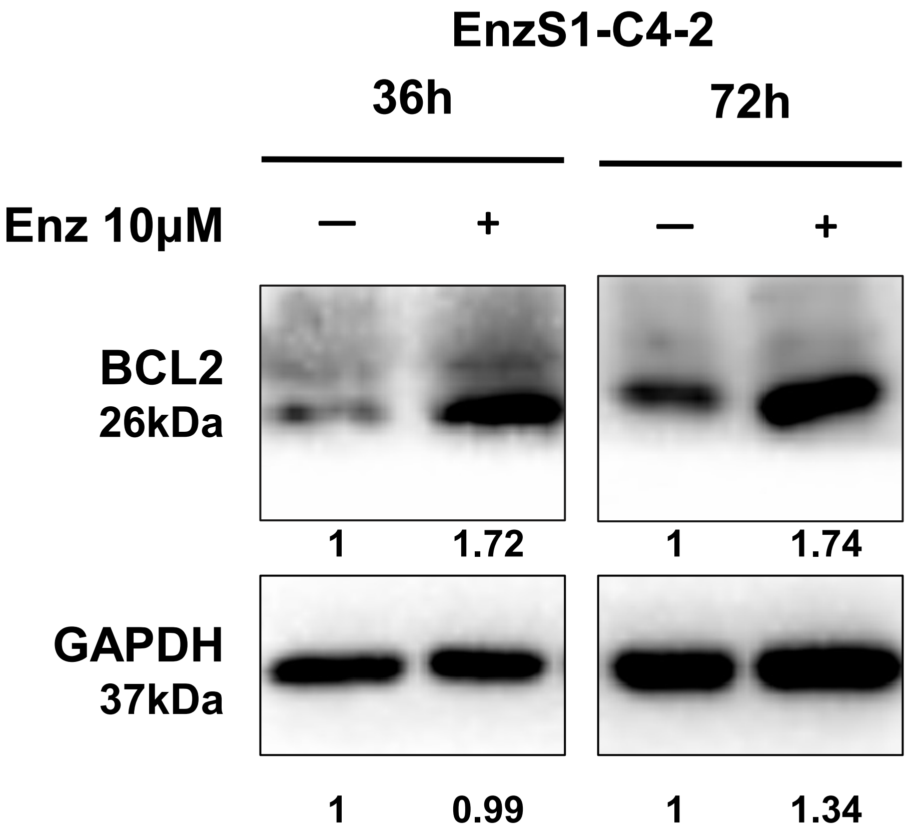
EnzS1-C4-2
36h
72h
Enz 10μM
—
+
—
+
BCL2
26kDa
1
1.72
1
1.74
GAPDH
37kDa
1
0.99
1
1.34

Figure 1D

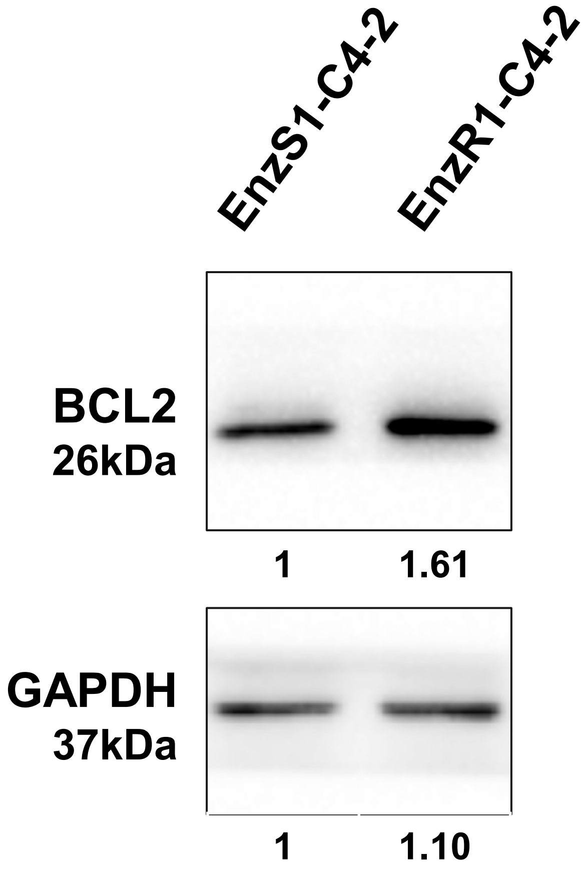

Figure 2A

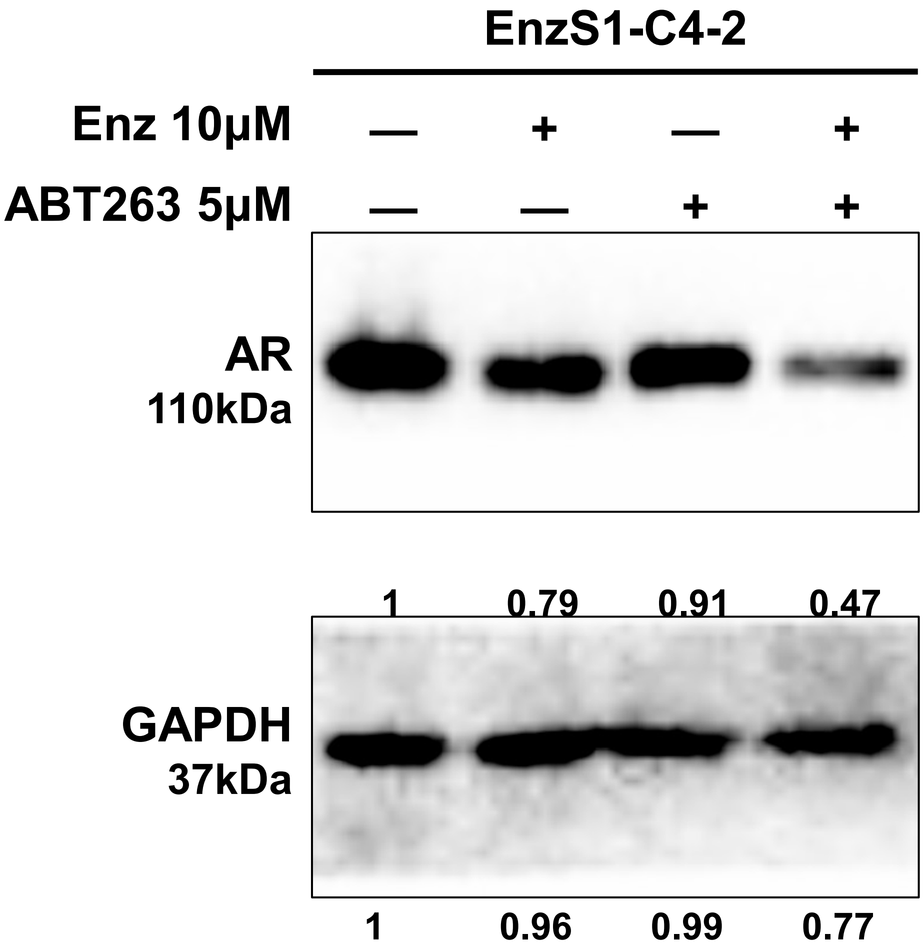
EnzS1-C4-2
Enz 10μM — + — +
ABT263 5μM — — + +
AR
110kDa
1 0.79 0.91 0.47
GAPDH
37kDa
1 0.96 0.99 0.77

Figure 2A

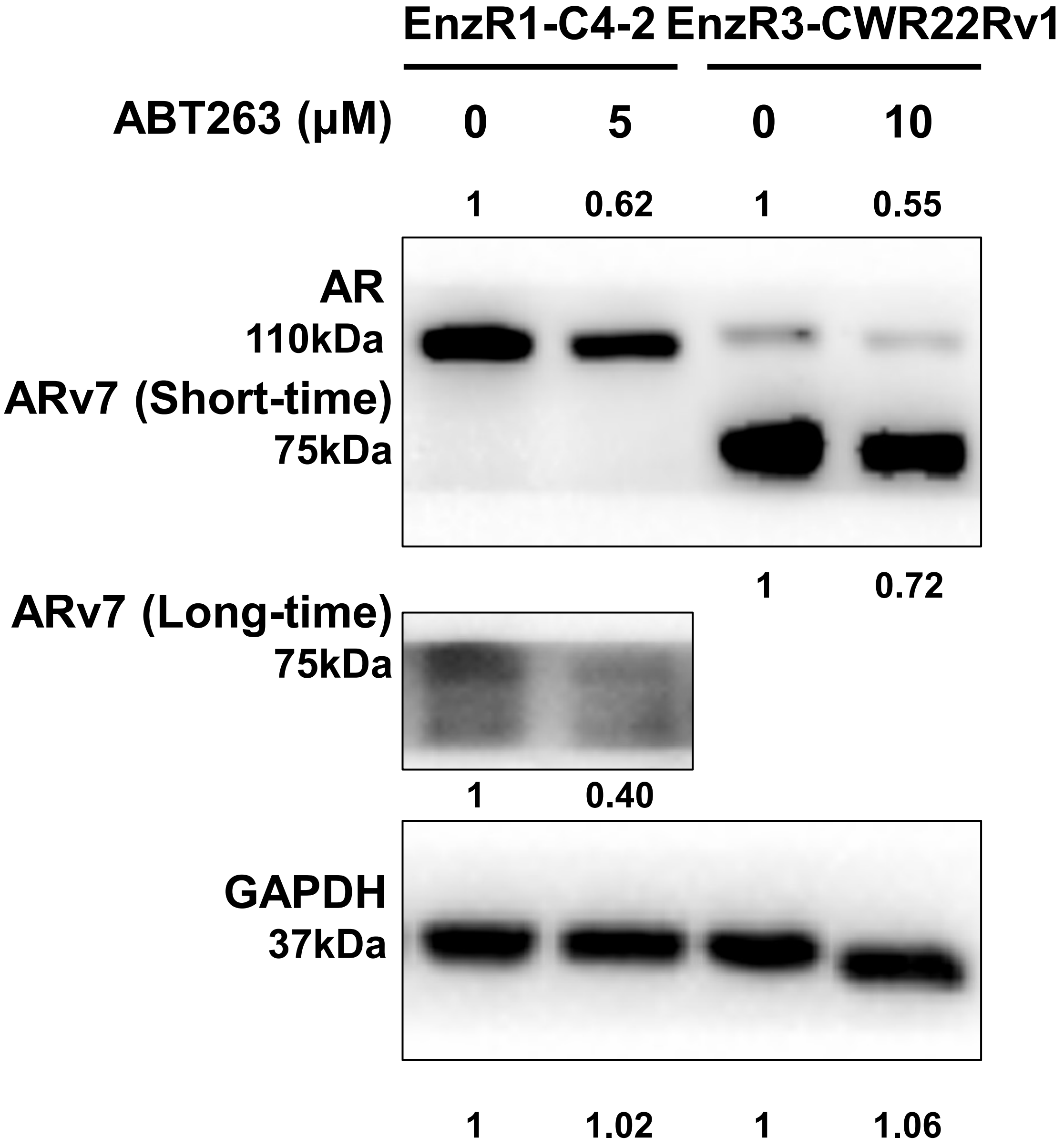
EnzR1-C4-2
EnzR3-CWR22Rv1
ABT263 (μM)
0
5
0
10
1
0.62
1
0.55
AR
110kDa
ARv7 (Short-time)
75kDa
1
0.72
ARv7 (Long-time)
75kDa
1
0.40
GAPDH
37kDa
1
1.02
1
1.06

Figure 2C

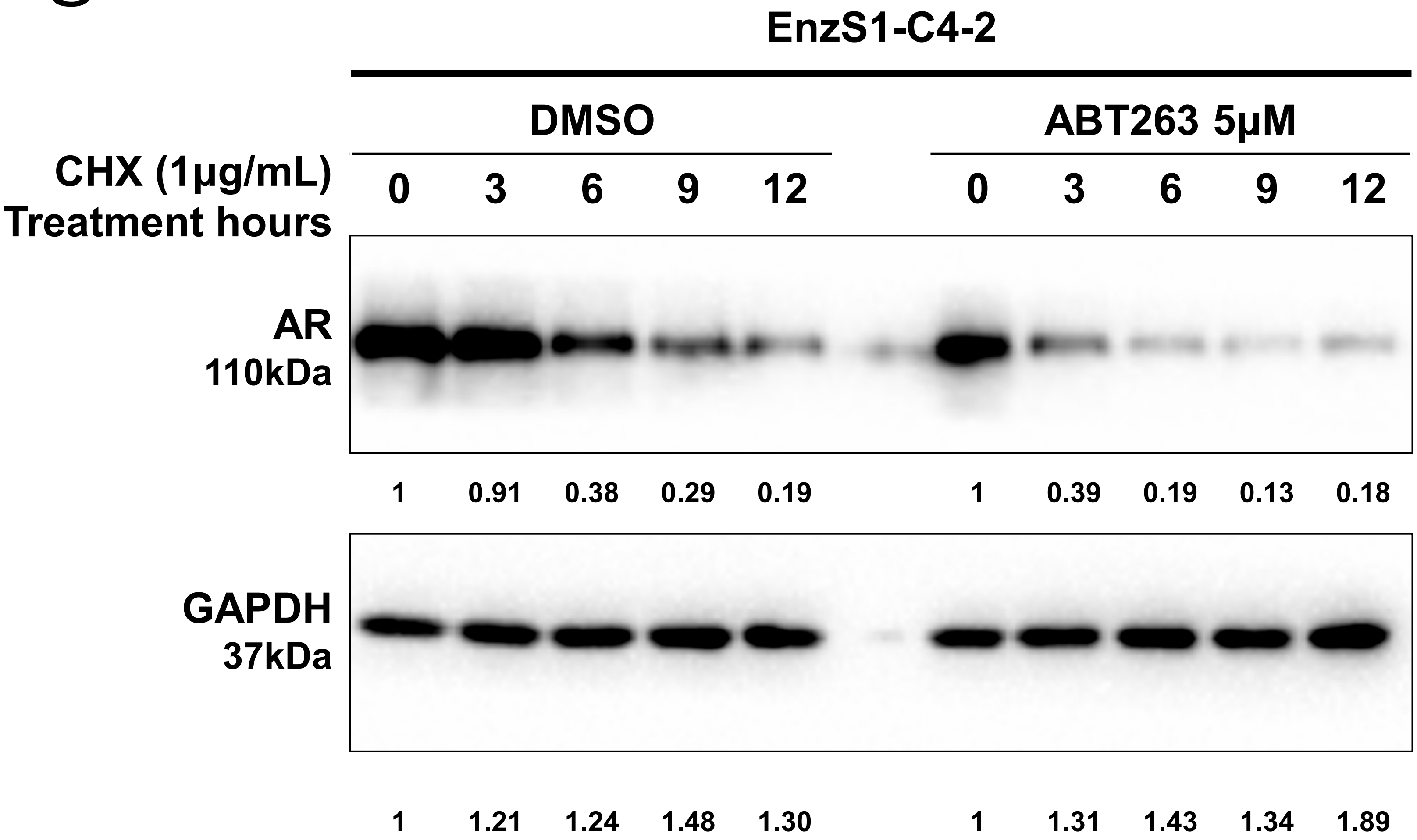

EnzS1-C4-2
DMSO
ABT263 5μM
CHX (1μg/mL)
Treatment hours
0 3 6 9 12
0 3 6 9 12
AR
110kDa
1 0.91 0.38 0.29 0.19
1 0.39 0.19 0.13 0.18
GAPDH
37kDa
1 1.21 1.24 1.48 1.30
1 1.31 1.43 1.34 1.89

Figure 2D

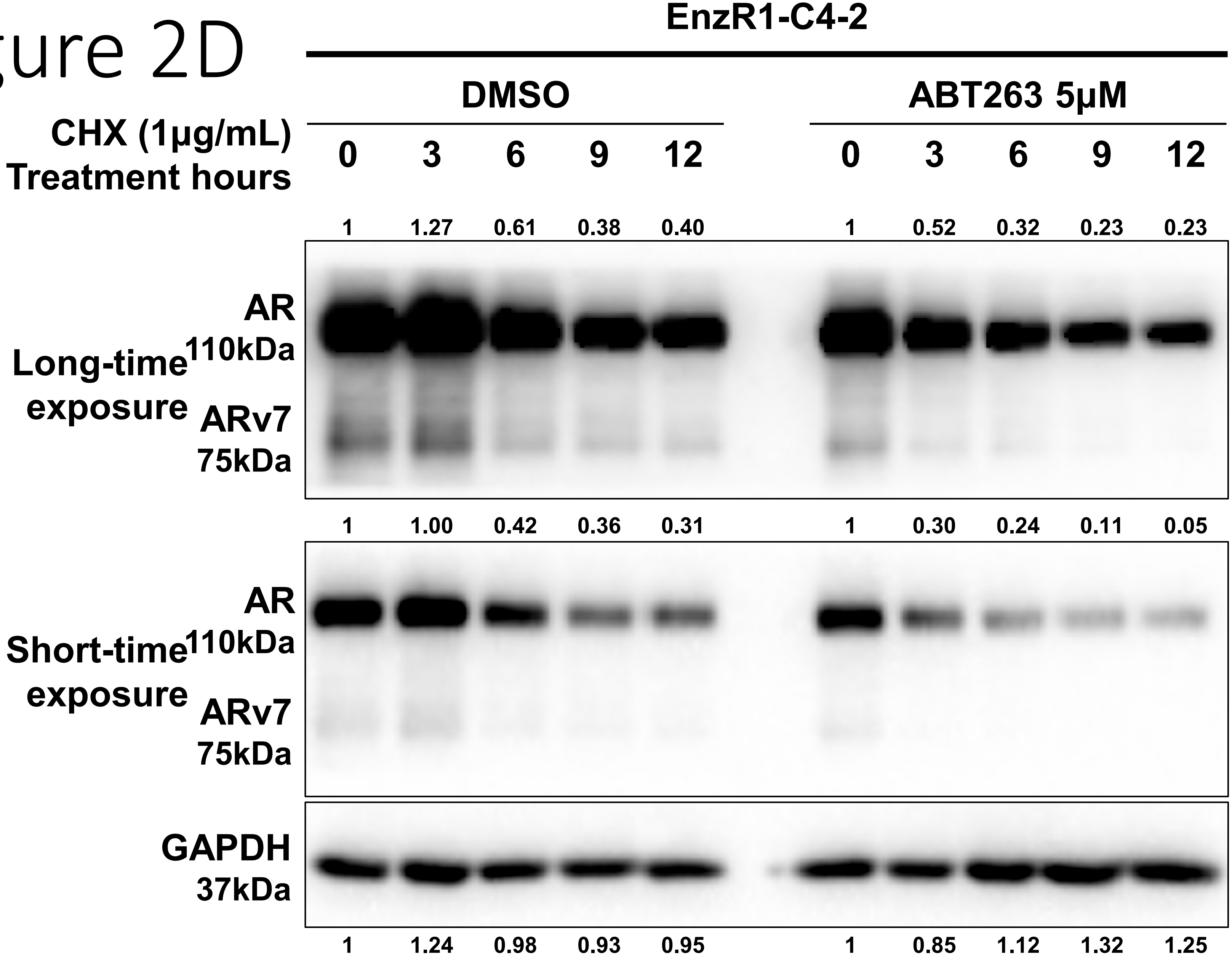
EnzR1-C4-2
DMSO
ABT263 5μM
CHX (1μg/mL)
Treatment hours
0 3 6 9 12
0 3 6 9 12
1 1.27 0.61 0.38 0.40
1 0.52 0.32 0.23 0.23
AR
110kDa
Long-time exposure
ARv7
75kDa
1 1.00 0.42 0.36 0.31
1 0.30 0.24 0.11 0.05
AR
110kDa
Short-time exposure
ARv7
75kDa
GAPDH
37kDa
1 1.24 0.98 0.93 0.95
1 0.85 1.12 1.32 1.25

Figure 2E

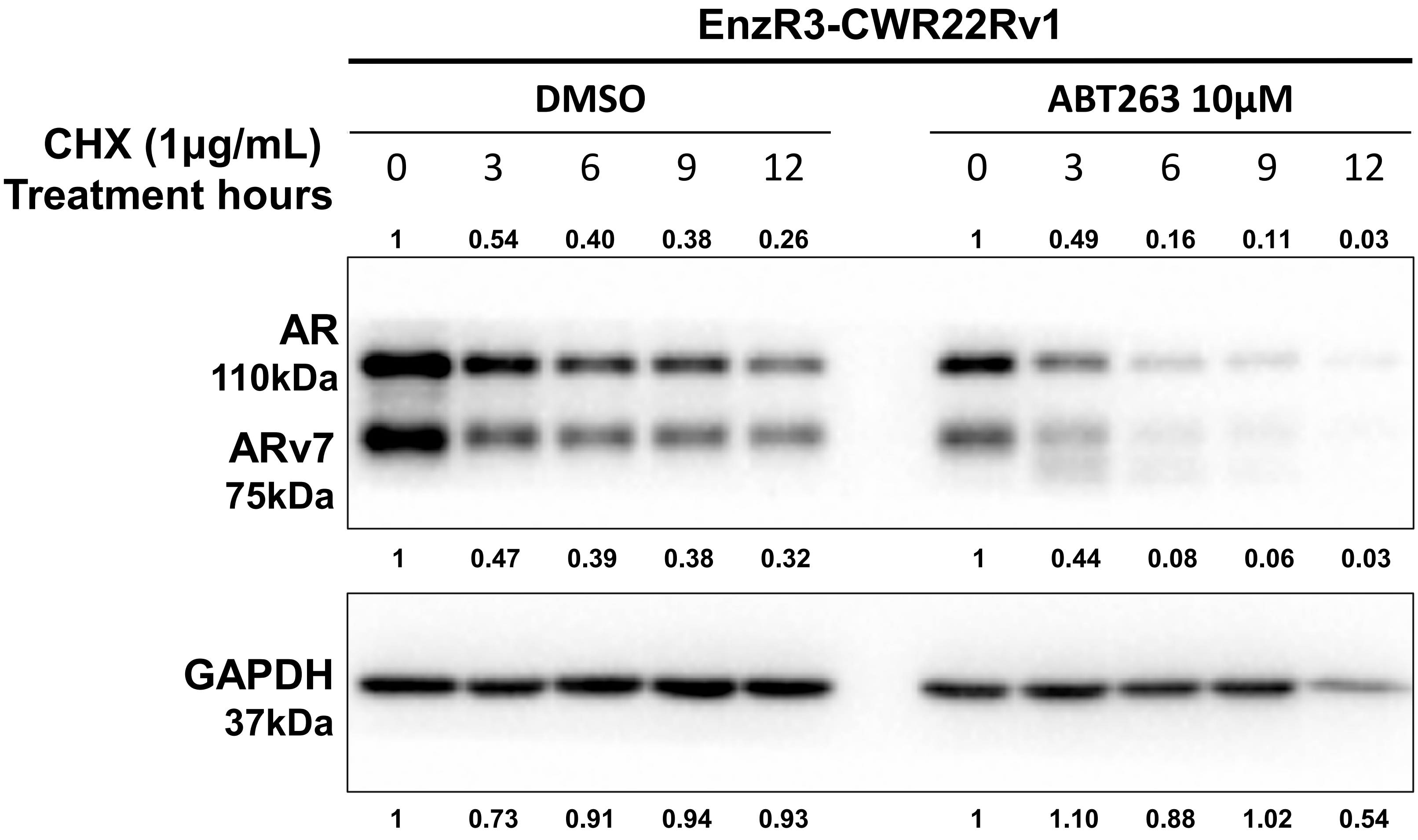
EnzR3-CWR22Rv1
DMSO
ABT263 10μM
CHX (1μg/mL)
Treatment hours
0 3 6 9 12
0 3 6 9 12
1 0.54 0.40 0.38 0.26
1 0.49 0.16 0.11 0.03
AR
110kDa
ARv7
75kDa
1 0.47 0.39 0.38 0.32
1 0.44 0.08 0.06 0.03
GAPDH
37kDa
1 0.73 0.91 0.94 0.93
1 1.10 0.88 1.02 0.54

Figure 2F

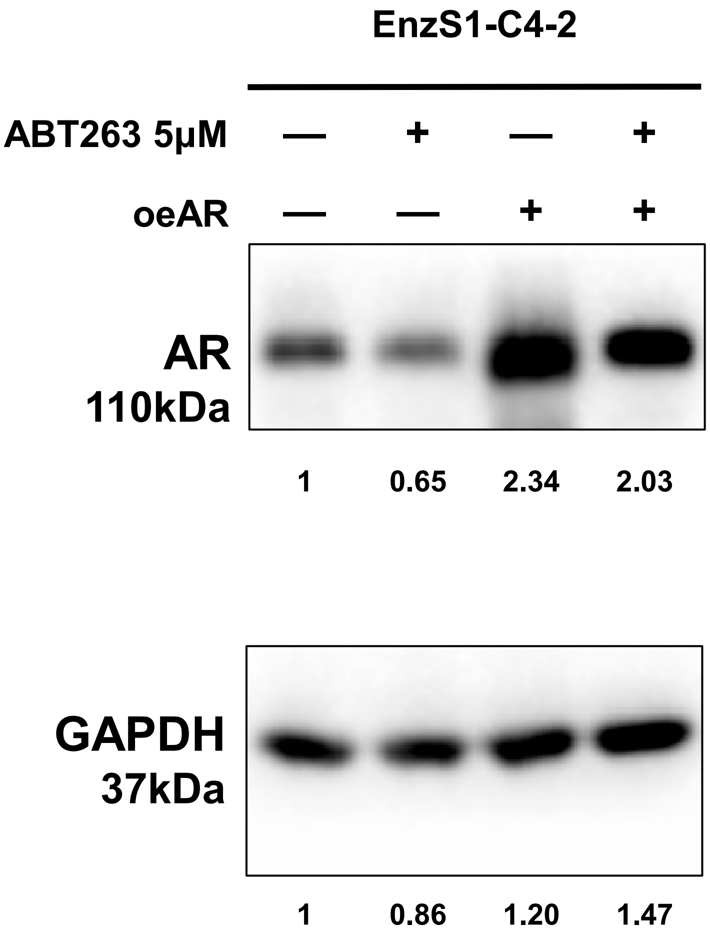
EnzS1-C4-2
ABT263 5μM — + — +
oeAR — — + +
AR
110kDa
1 0.65 2.34 2.03
GAPDH
37kDa
1 0.86 1.20 1.47

Figure 2G

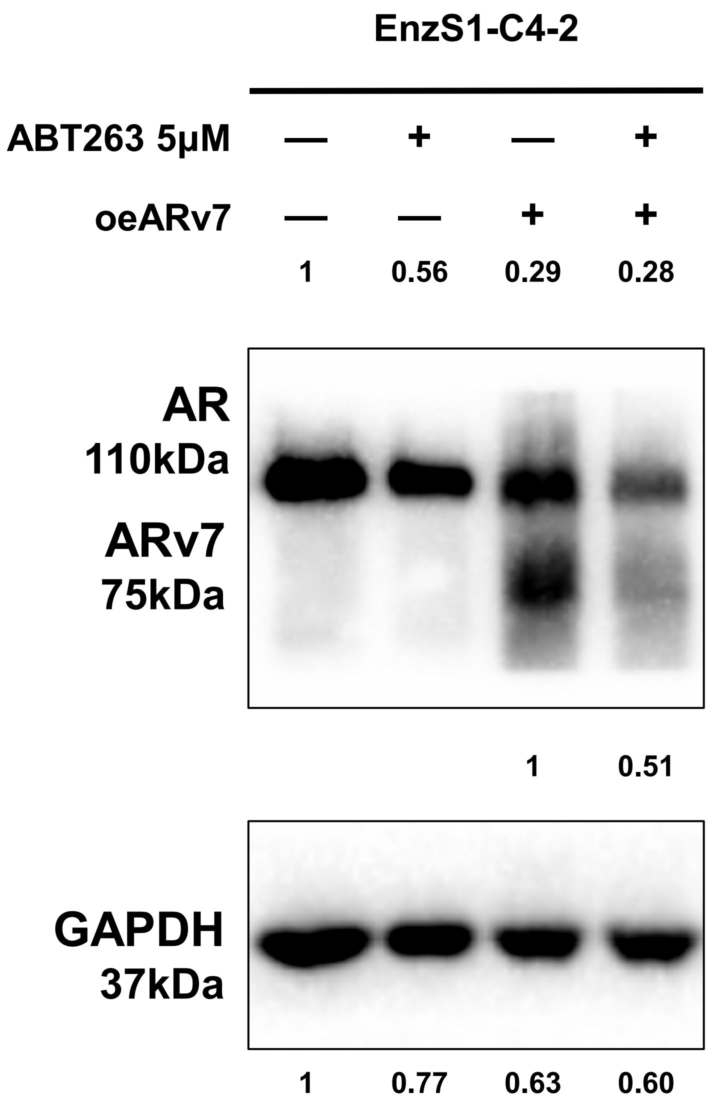

EnzS1-C4-2
ABT263 5μM — + — +
oeARv7 — — + +
1 0.56 0.29 0.28
AR
110kDa
ARv7
75kDa
1 0.51
GAPDH
37kDa
1 0.77 0.63 0.60

Figure 2H

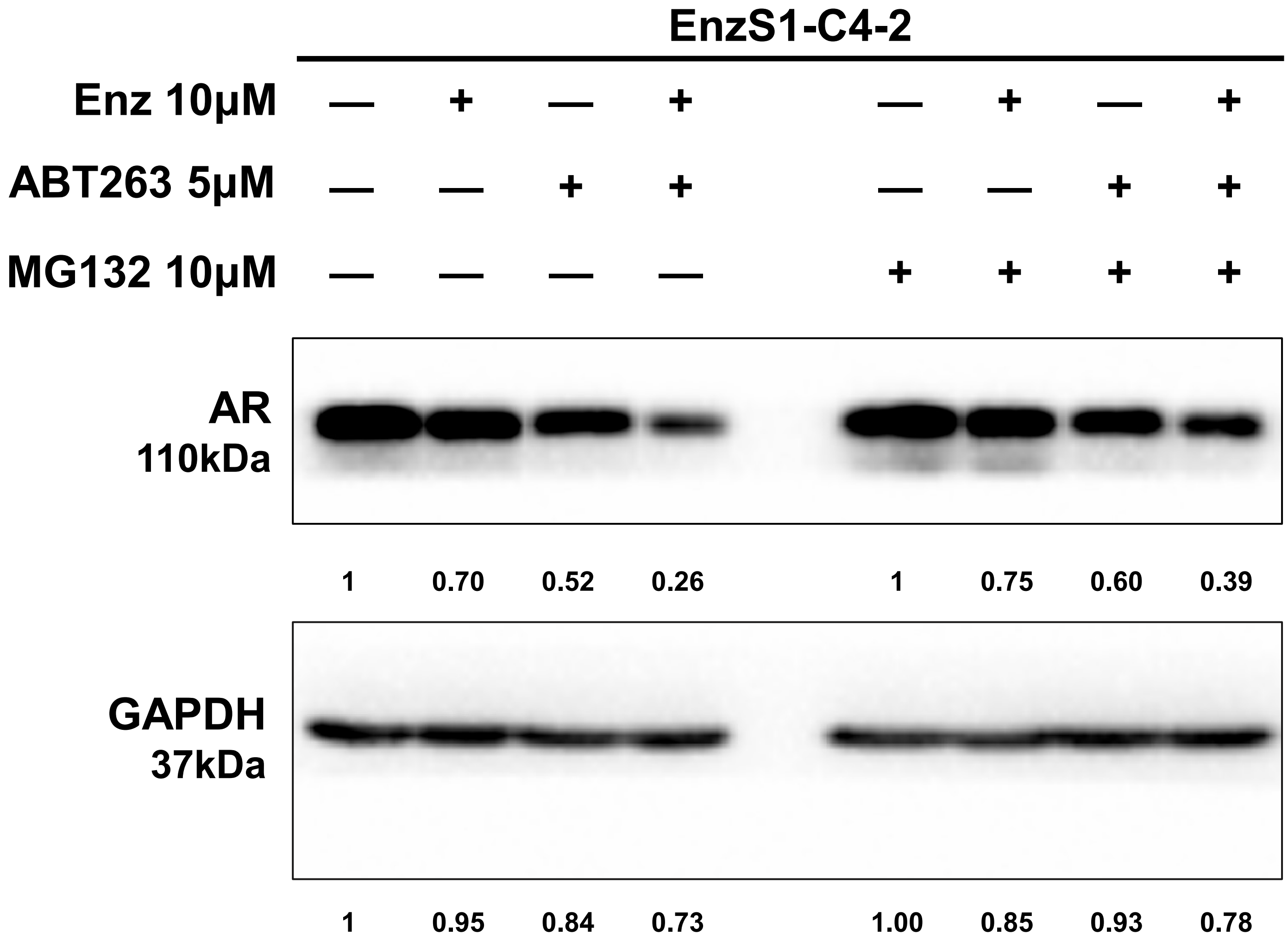

EnzS1-C4-2
Enz 10μM — + — + — + — +
ABT263 5μM — — + + — — + +
MG132 10μM — — — — + + + +
AR
110kDa
1 0.70 0.52 0.26 1 0.75 0.60 0.39
GAPDH
37kDa
1 0.95 0.84 0.73 1.00 0.85 0.93 0.78

Figure 2H

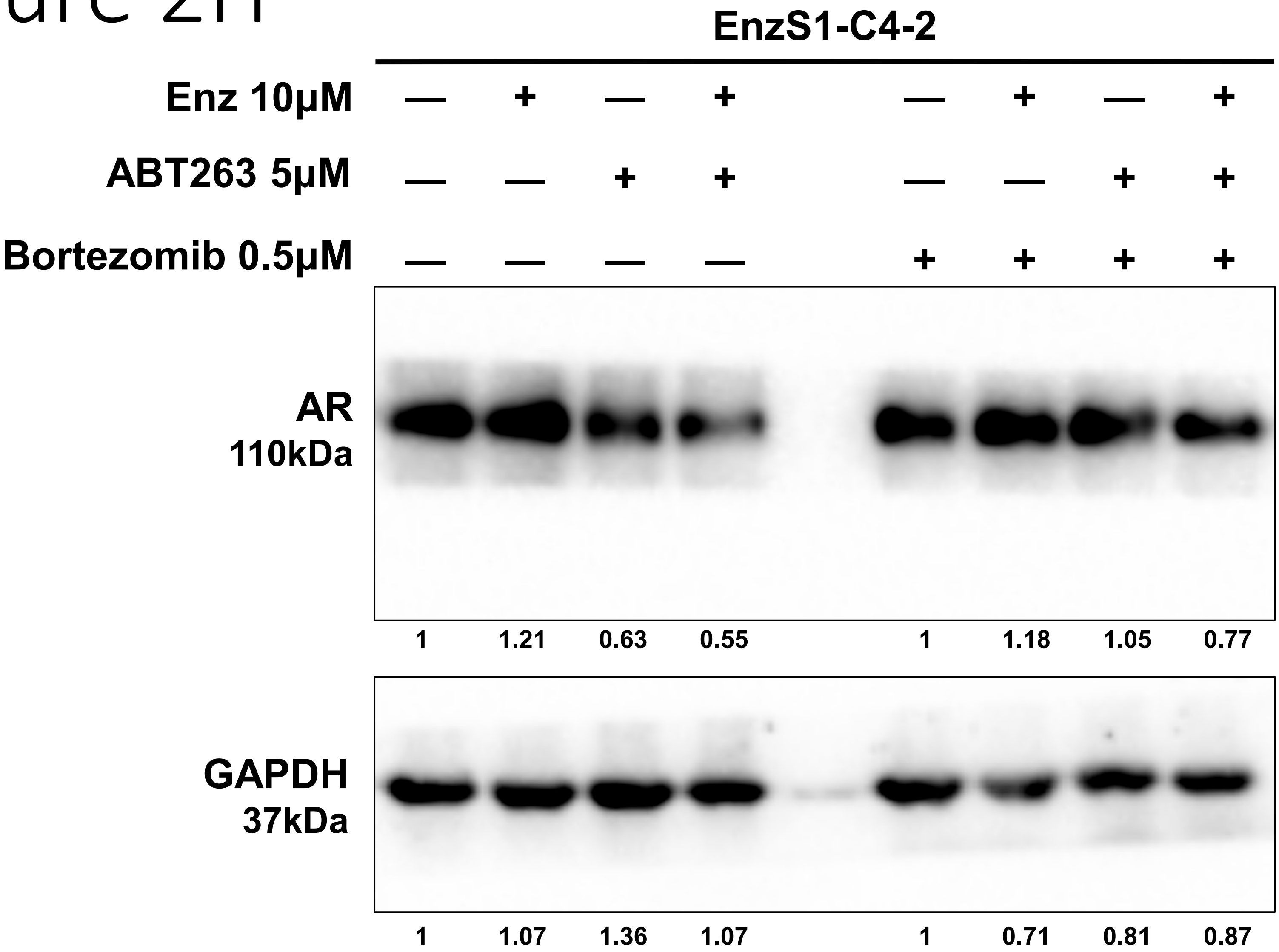
EnzS1-C4-2
Enz 10μM — + — + — + — +
ABT263 5μM — — + + — — + +
Bortezomib 0.5μM — — — — + + + +
AR
110kDa
1 1.21 0.63 0.55 1 1.18 1.05 0.77
GAPDH
37kDa
1 1.07 1.36 1.07 1 0.71 0.81 0.87

Figure 2I

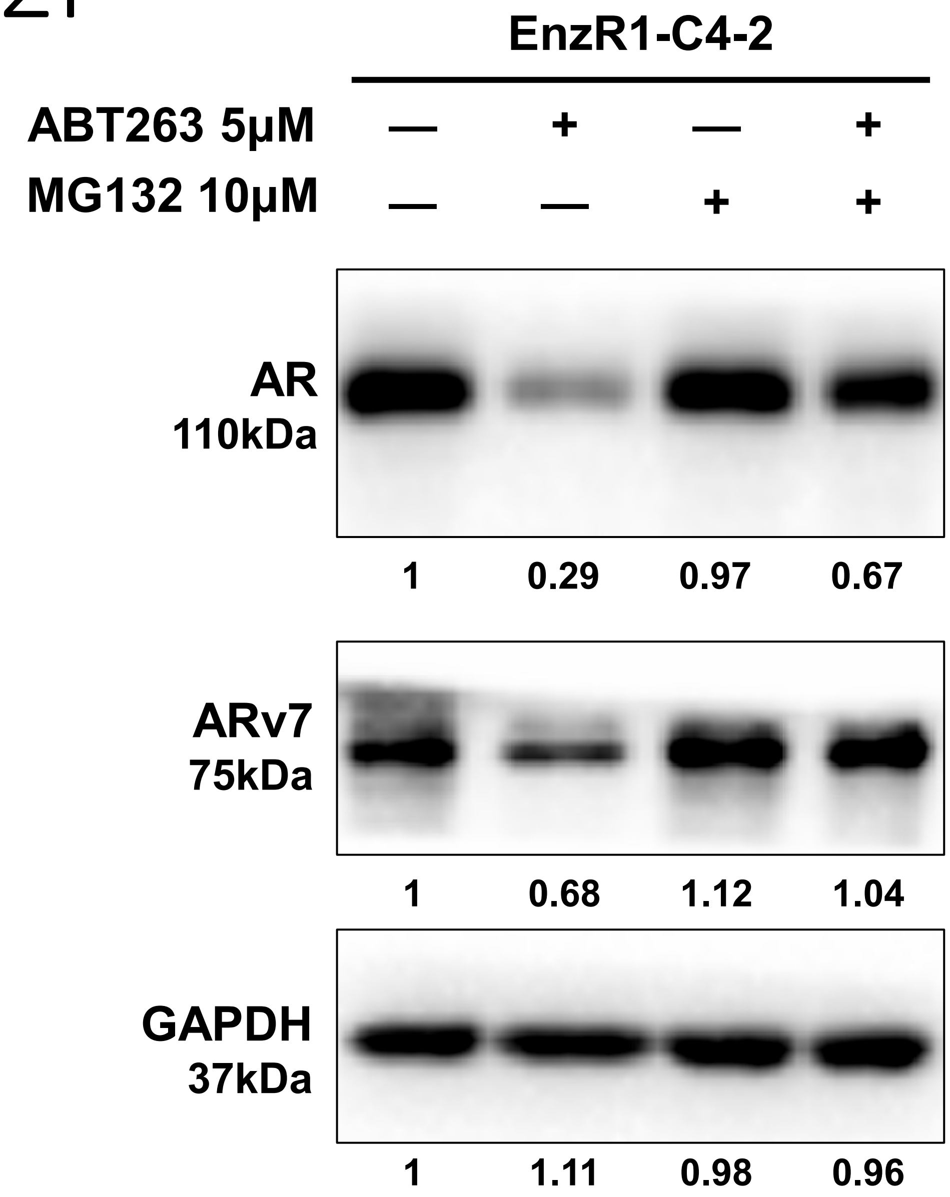

EnzR1-C4-2
ABT263 5μM — + — +
MG132 10μM — — + +
AR
110kDa
1 0.29 0.97 0.67
ARv7
75kDa
1 0.68 1.12 1.04
GAPDH
37kDa
1 1.11 0.98 0.96

Figure 2I

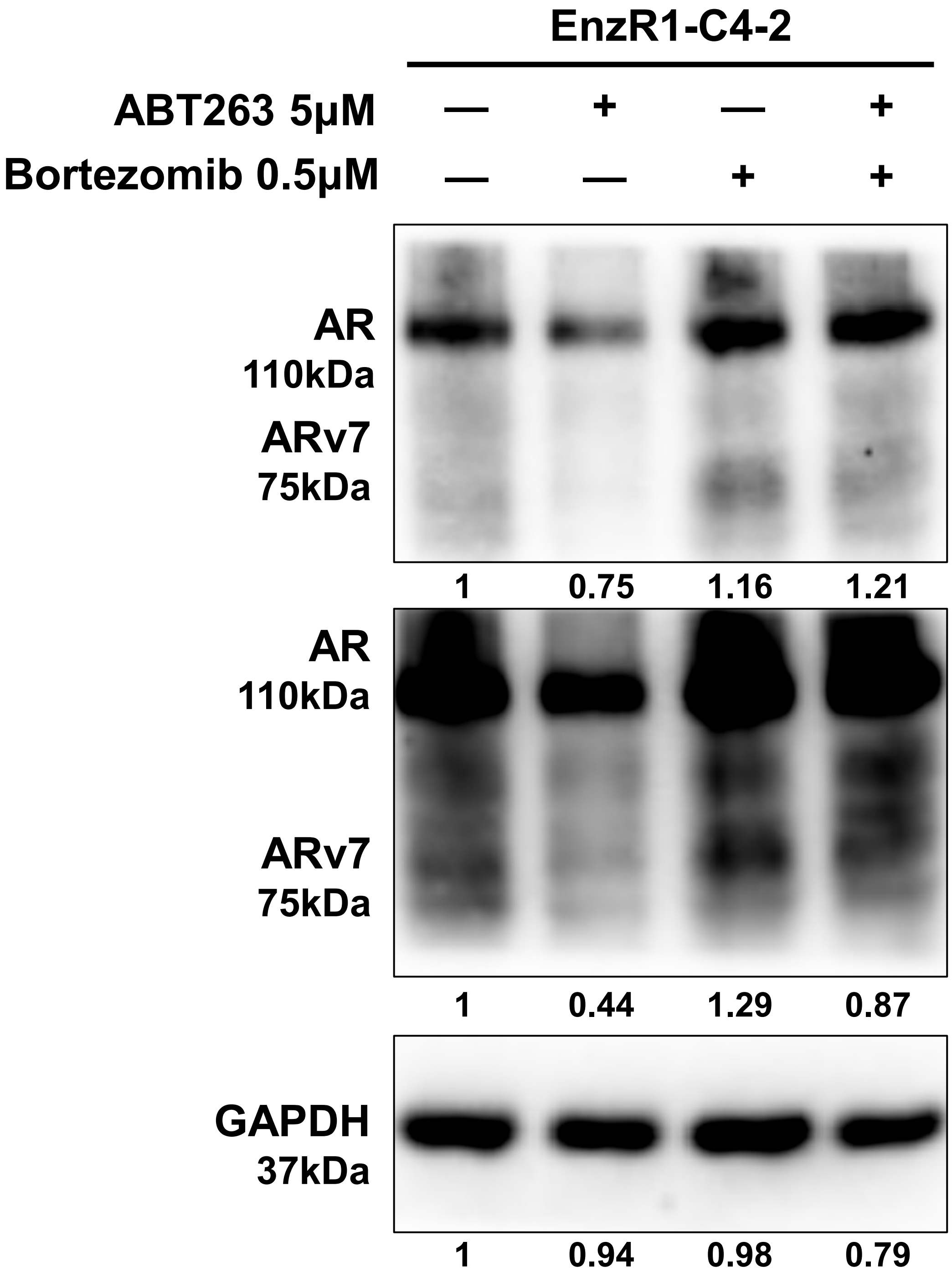

EnzR1-C4-2
ABT263 5μM — + — +
Bortezomib 0.5μM — — + +
AR
110kDa
ARv7
75kDa
1 0.75 1.16 1.21
AR
110kDa
ARv7
75kDa
1 0.44 1.29 0.87
GAPDH
37kDa
1 0.94 0.98 0.79

Figure 2J

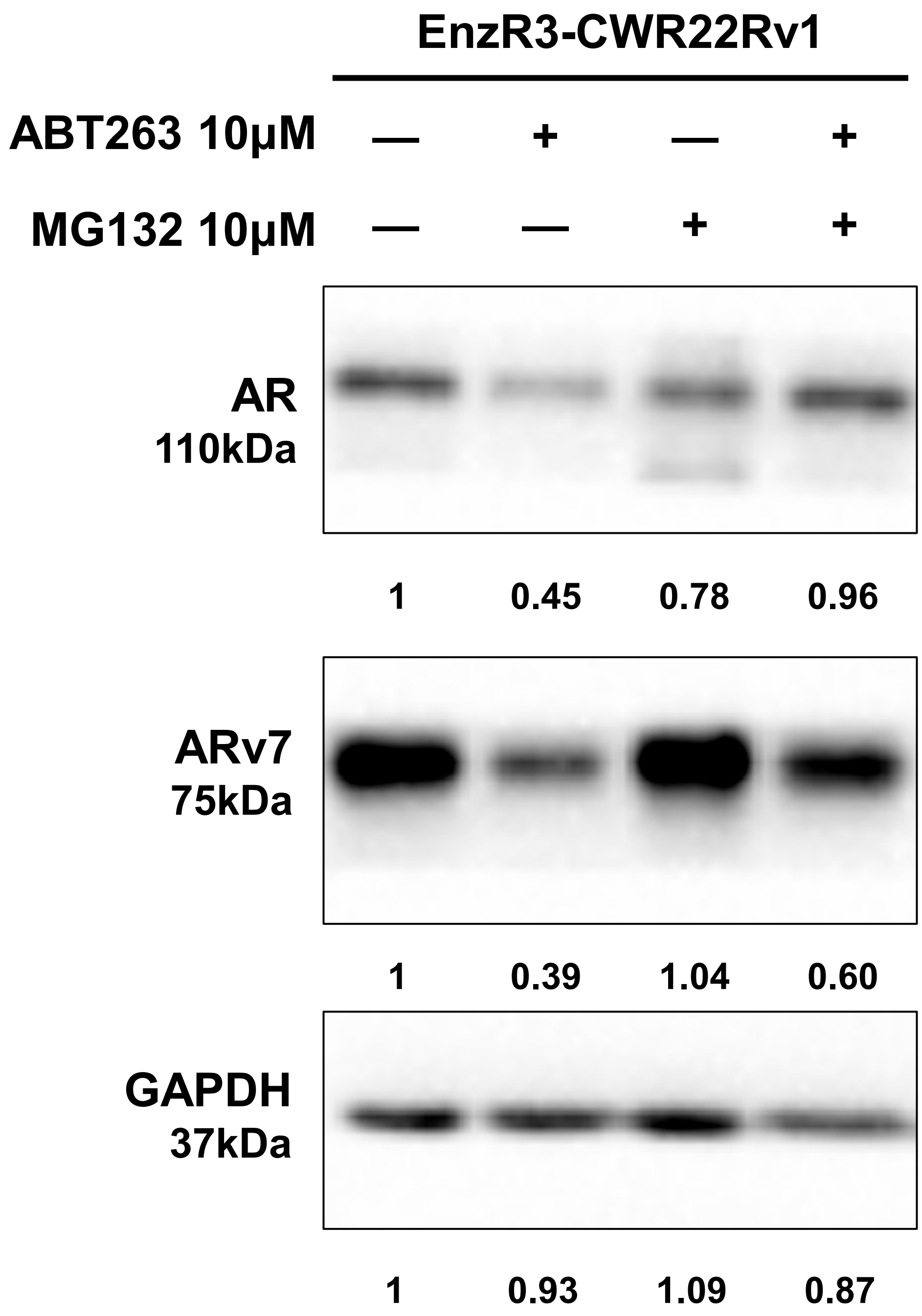
EnzR3-CWR22Rv1
ABT263 10μM — + — +
MG132 10μM — — + +
AR
110kDa
1 0.45 0.78 0.96
ARv7
75kDa
1 0.39 1.04 0.60
GAPDH
37kDa
1 0.93 1.09 0.87

Figure 2J

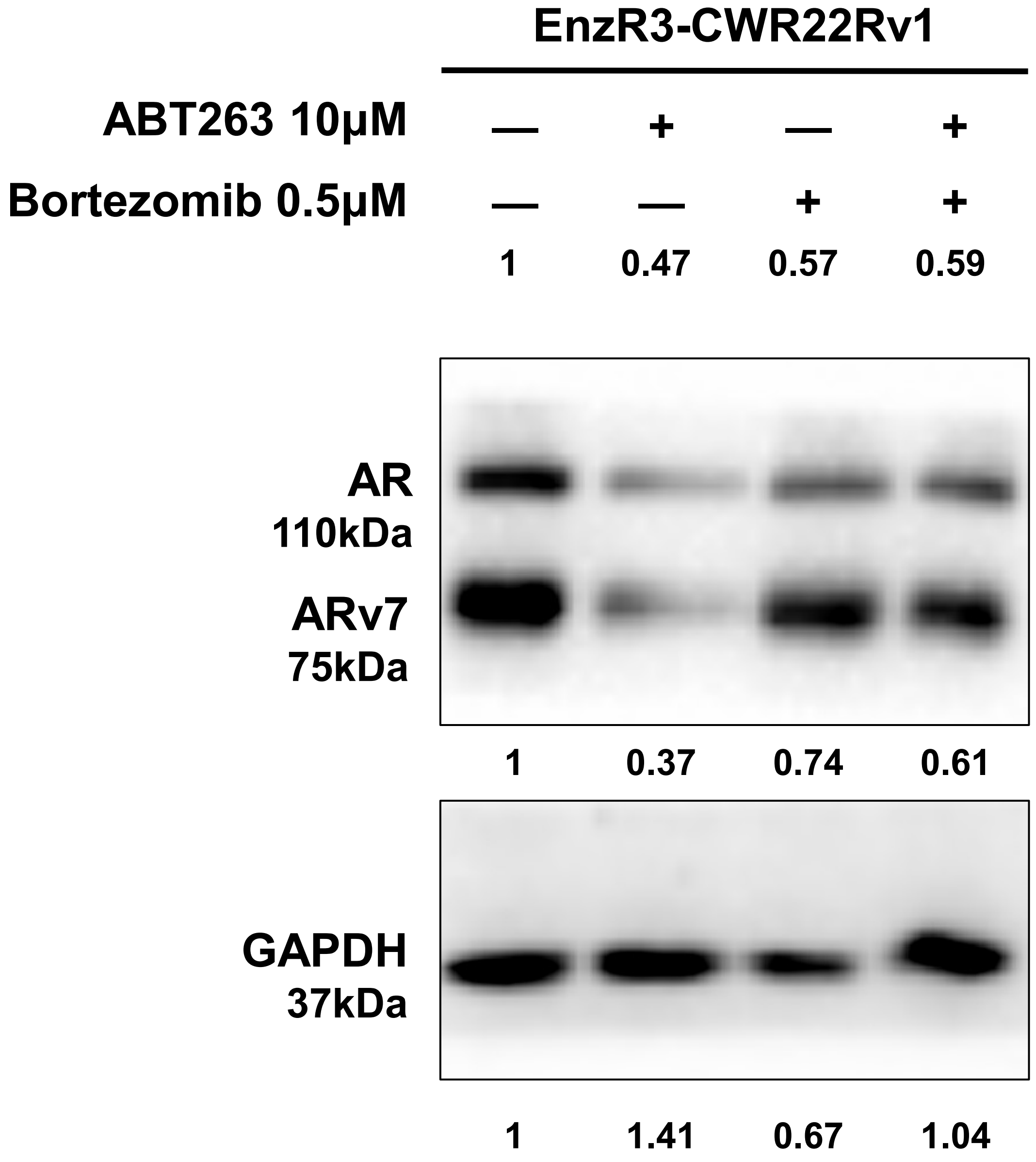
EnzR3-CWR22Rv1
ABT263 10μM — + — +
Bortezomib 0.5μM — — + +
1 0.47 0.57 0.59
AR
110kDa
ARv7
75kDa
1 0.37 0.74 0.61
GAPDH
37kDa
1 1.41 0.67 1.04

Figure 2K

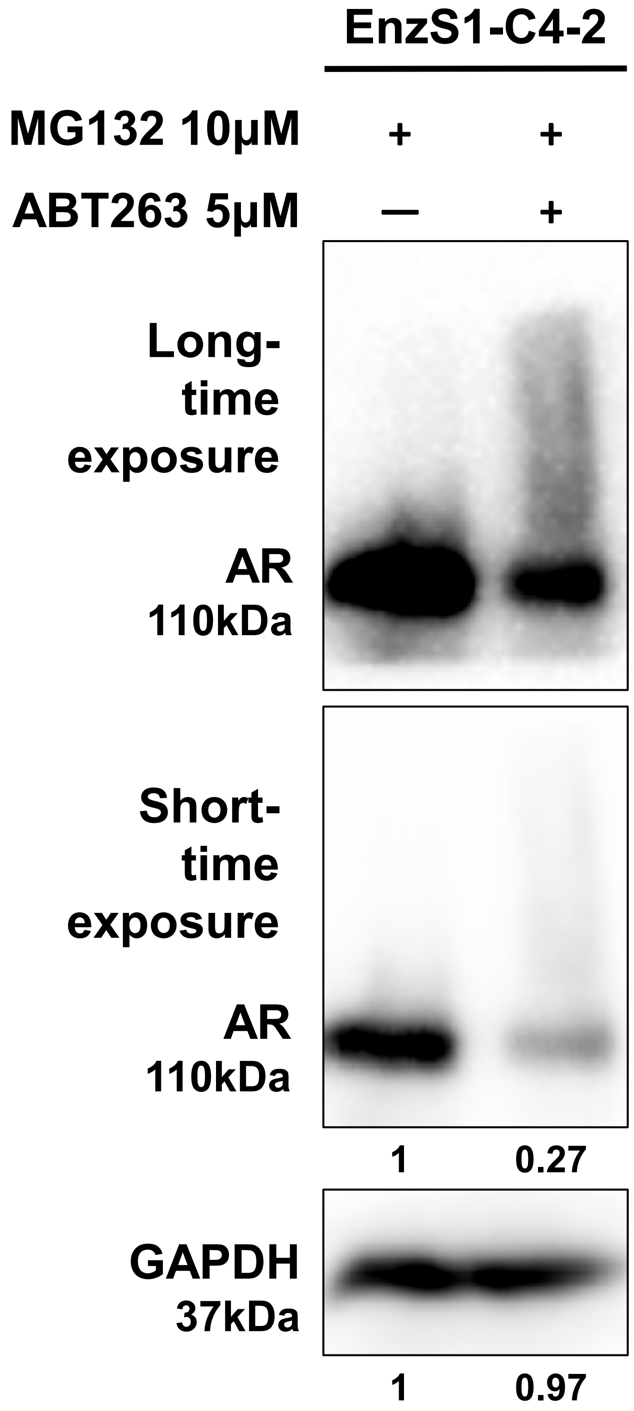
EnzS1-C4-2
MG132 10μM + +
ABT263 5μM — +
Long-time exposure
AR 110kDa
Short-time exposure
AR 110kDa
1 0.27
GAPDH 37kDa
1 0.97

Figure 2L

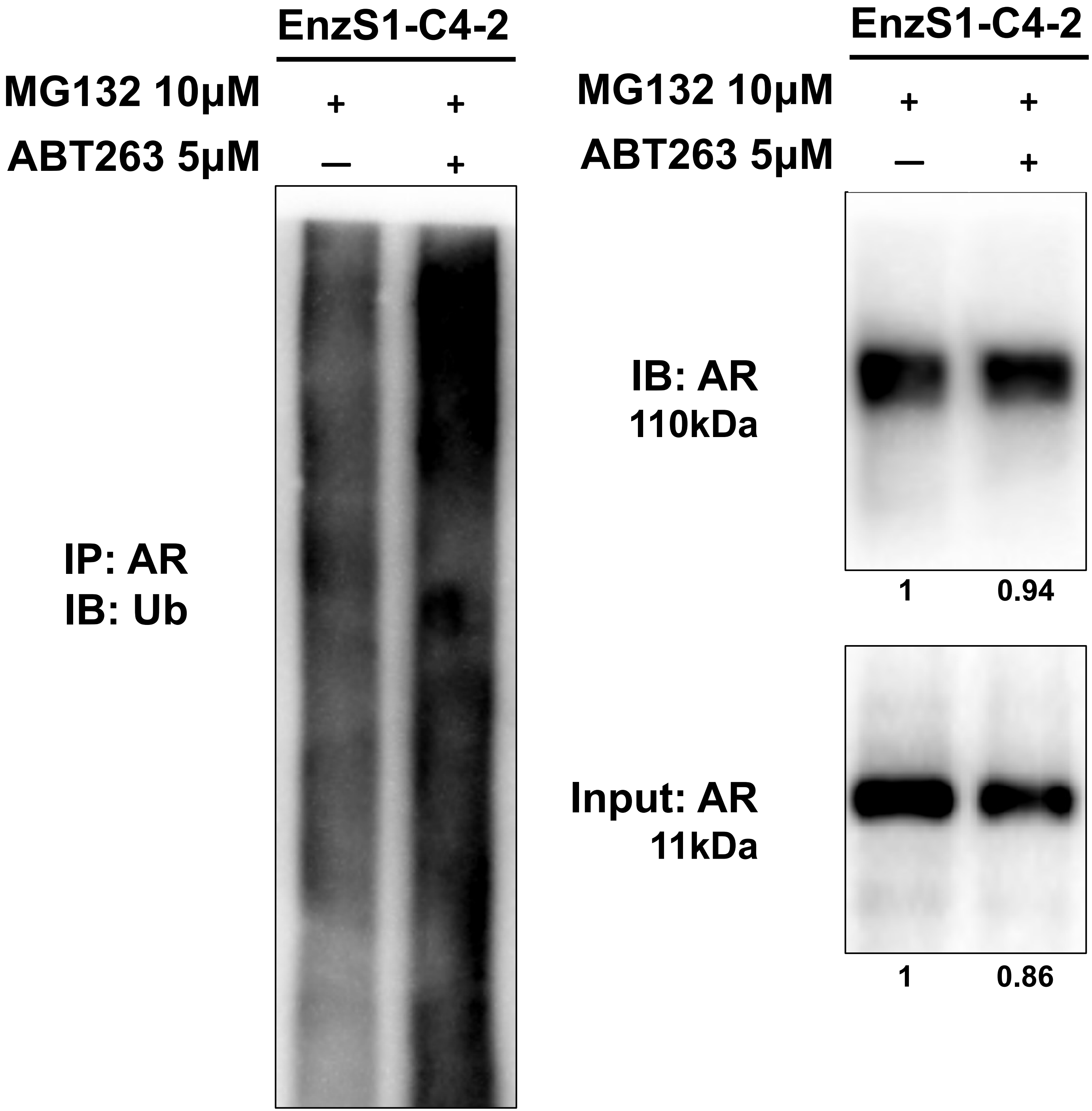
EnzS1-C4-2
MG132 10μM + +
ABT263 5μM — +
IP: AR
IB: Ub
EnzS1-C4-2
MG132 10μM + +
ABT263 5μM — +
IB: AR
110kDa
1 0.94
Input: AR
11kDa
1 0.86

Figure 2M

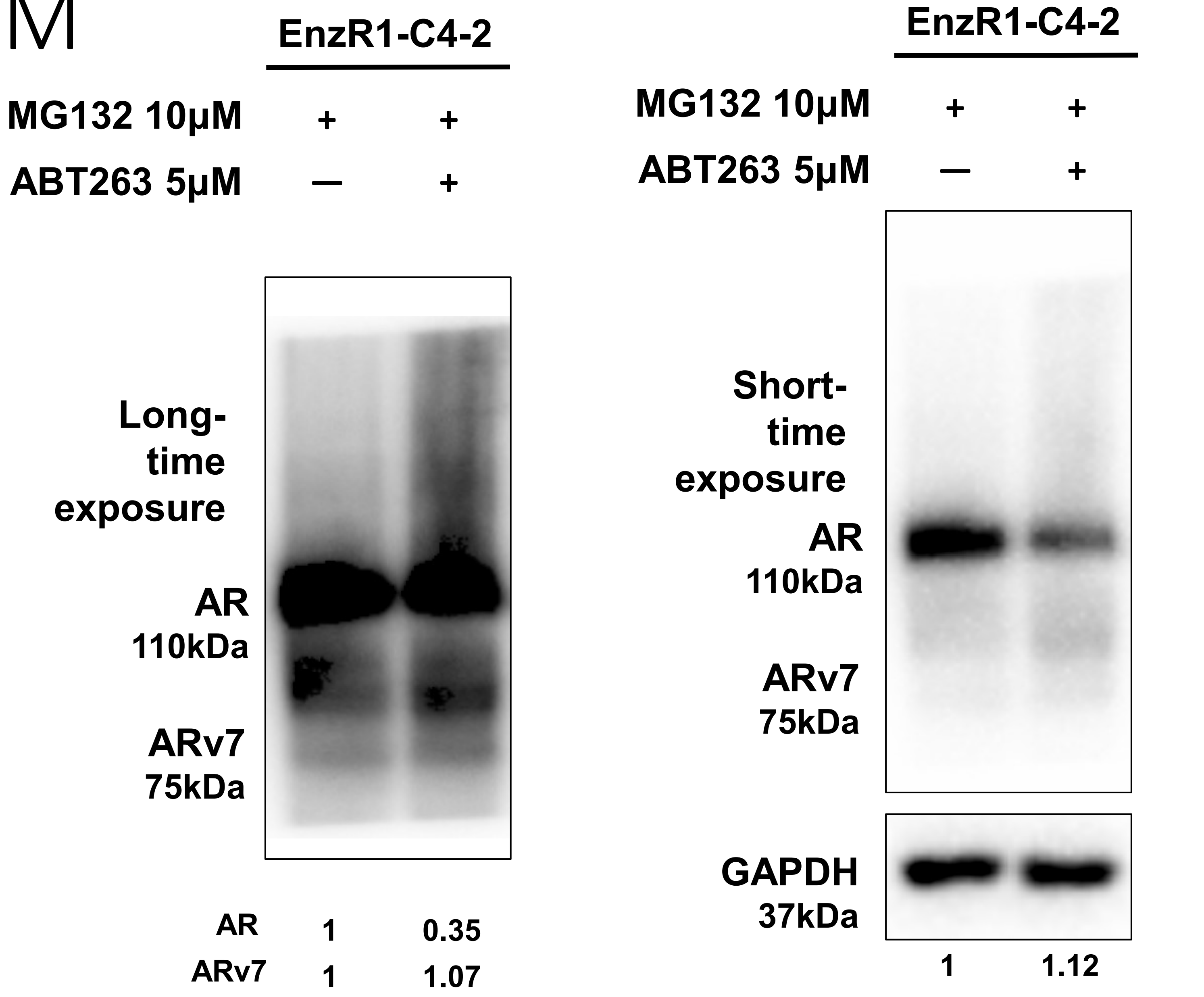
EnzR1-C4-2
MG132 10μM + +
ABT263 5μM — +
Long-time exposure
AR 110kDa
ARv7 75kDa
AR 1 0.35
ARv7 1 1.07
EnzR1-C4-2
MG132 10μM + +
ABT263 5μM — +
Short-time exposure
AR 110kDa
ARv7 75kDa
GAPDH 37kDa
1 1.12

Figure 2N

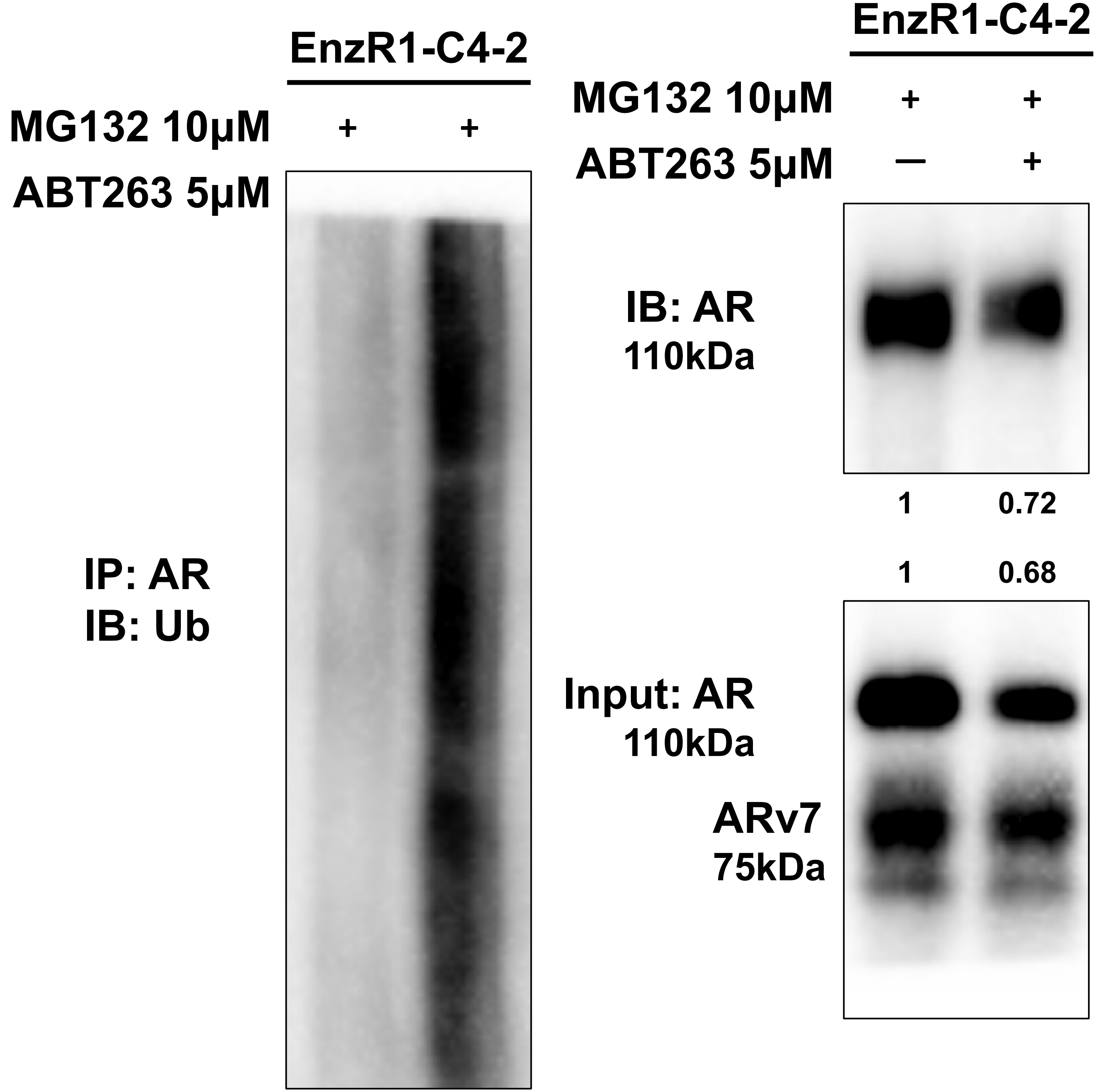
EnzR1-C4-2
MG132 10μM + +
ABT263 5μM
IP: AR
IB: Ub
EnzR1-C4-2
MG132 10μM + +
ABT263 5μM — +
IB: AR
110kDa
1 0.72
1 0.68
Input: AR
110kDa
ARv7
75kDa

Figure 4A

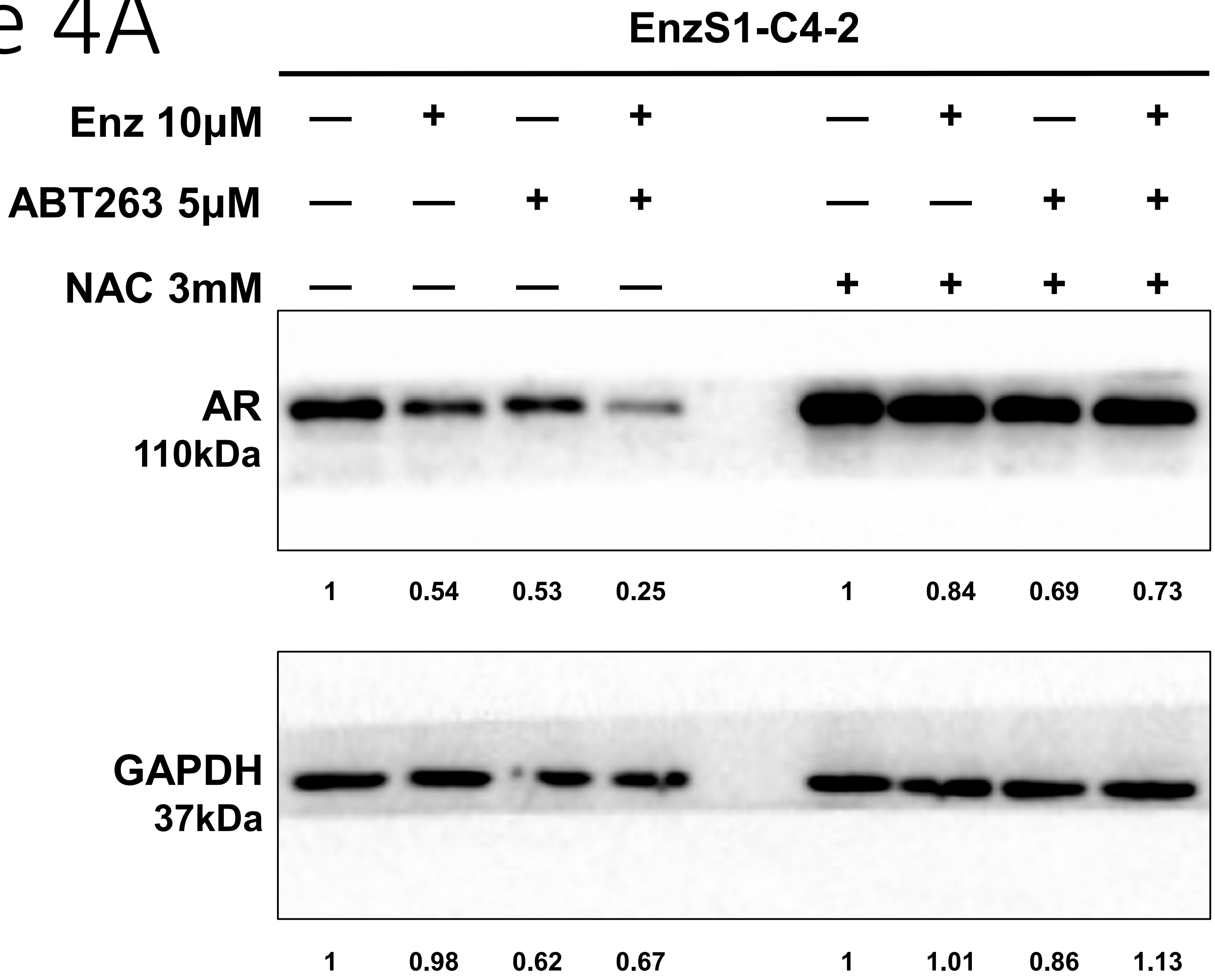
EnzS1-C4-2
Enz 10μM — + — + — + — +
ABT263 5μM — — + + — — + +
NAC 3mM — — — — + + + +
AR
110kDa
1 0.54 0.53 0.25 1 0.84 0.69 0.73
GAPDH
37kDa
1 0.98 0.62 0.67 1 1.01 0.86 1.13

Figure 4B

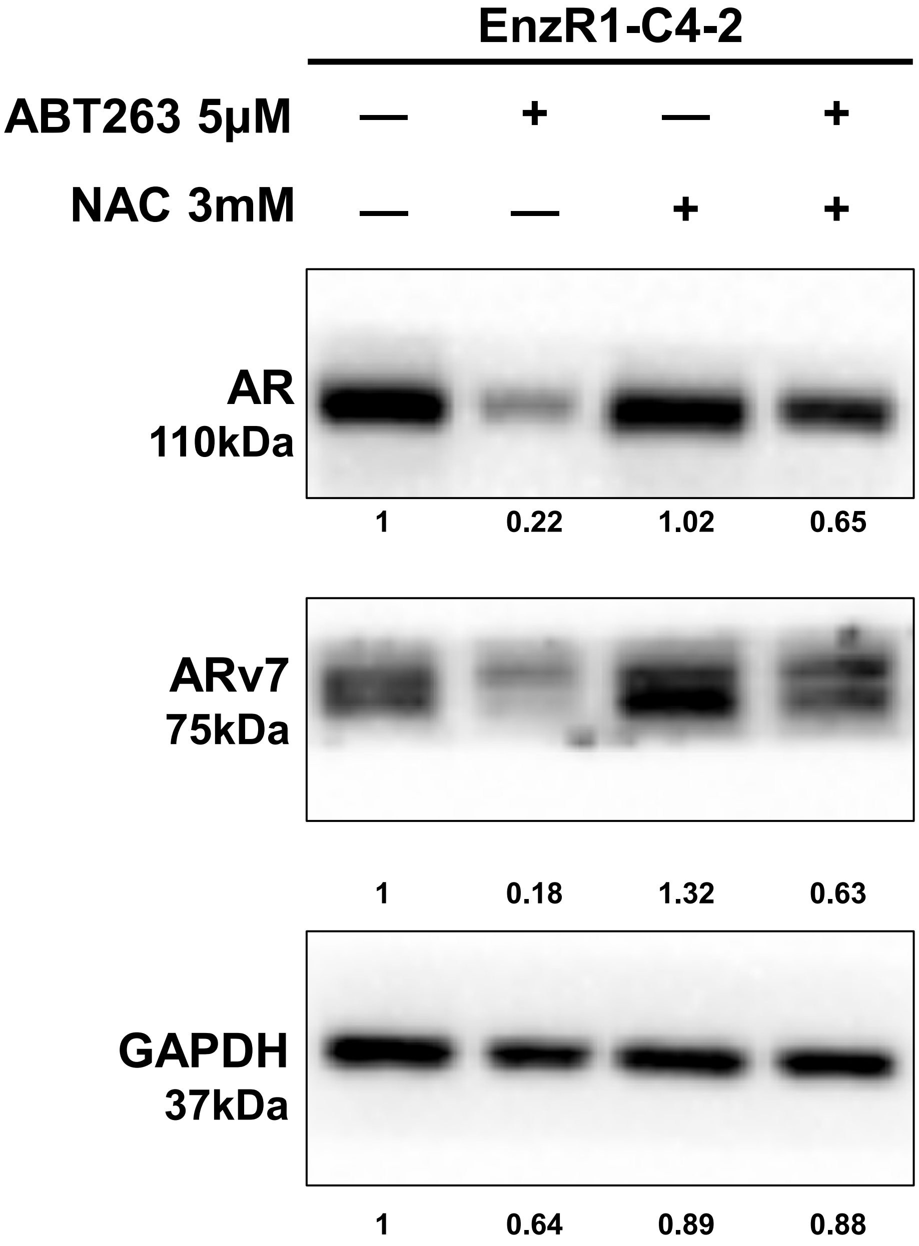

EnzR1-C4-2
ABT263 5μM — + — +
NAC 3mM — — + +
AR
110kDa
1 0.22 1.02 0.65
ARv7
75kDa
1 0.18 1.32 0.63
GAPDH
37kDa
1 0.64 0.89 0.88

Figure 4C

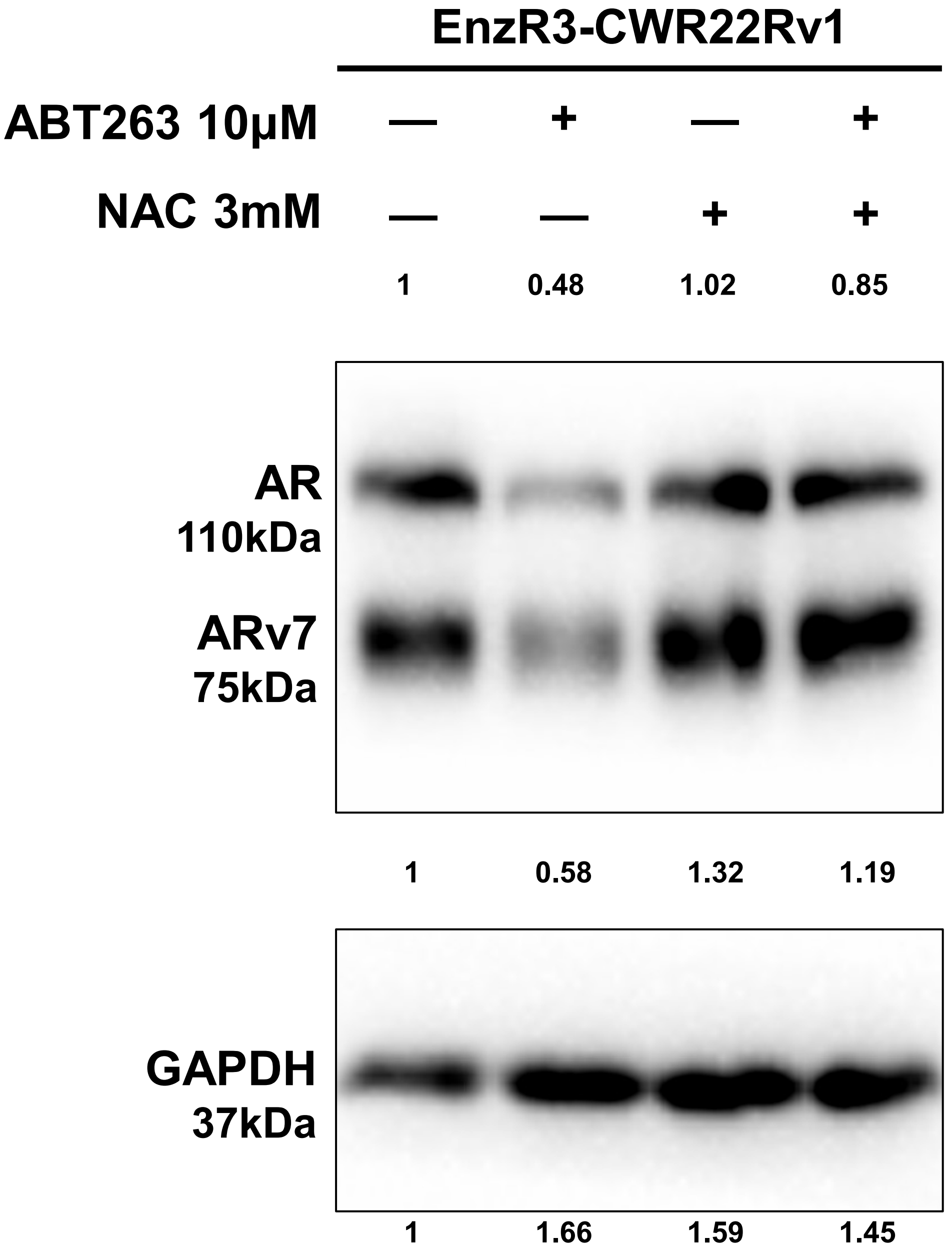
EnzR3-CWR22Rv1
ABT263 10μM — + — +
NAC 3mM — — + +
1 0.48 1.02 0.85
AR
110kDa
ARv7
75kDa
1 0.58 1.32 1.19
GAPDH
37kDa
1 1.66 1.59 1.45

Figure 4D

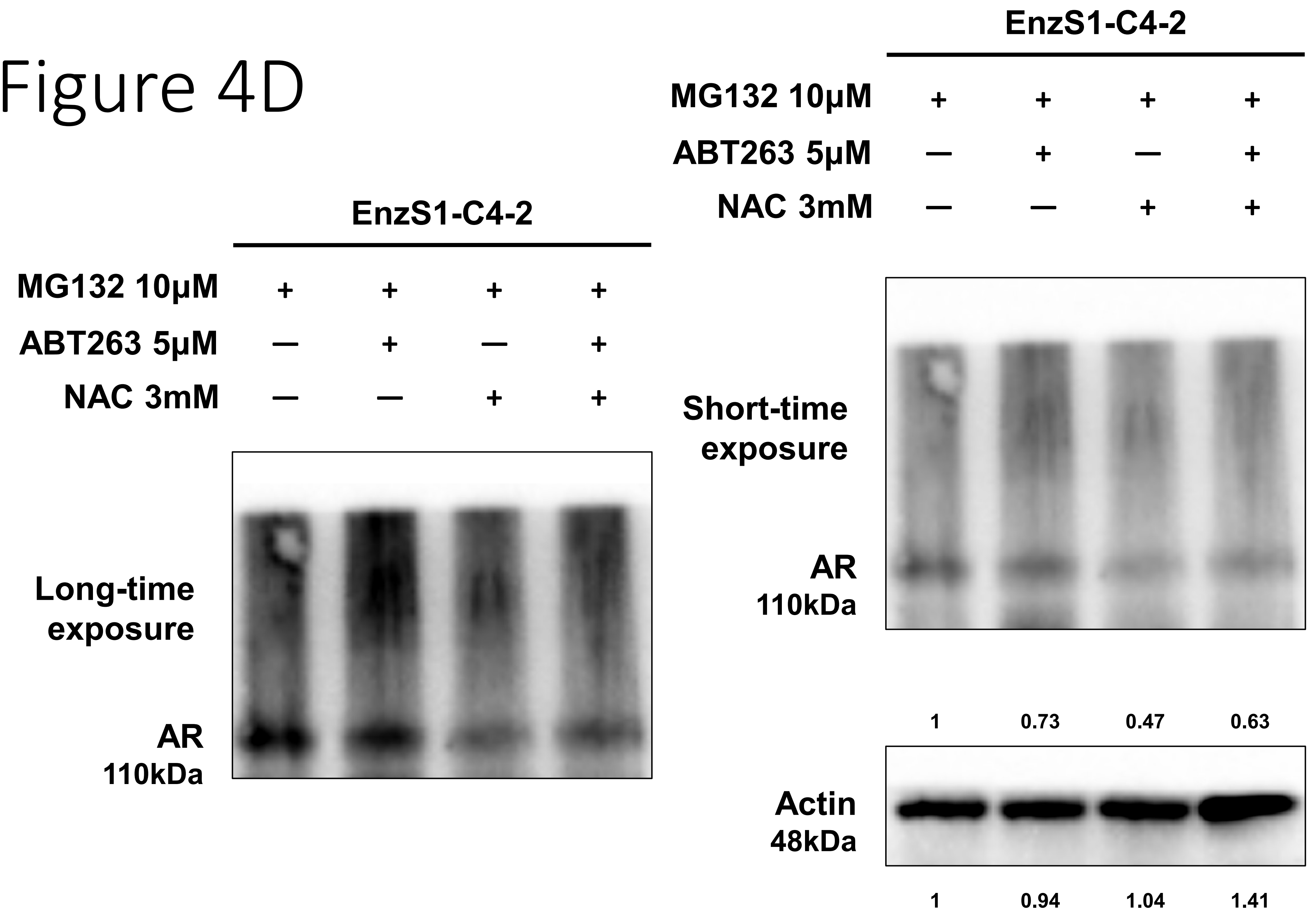

EnzS1-C4-2
MG132 10μM + + + +
ABT263 5μM — + — +
NAC 3mM — — + +
Long-time exposure
AR
110kDa
EnzS1-C4-2
MG132 10μM + + + +
ABT263 5μM — + — +
NAC 3mM — — + +
Short-time exposure
AR
110kDa
1 0.73 0.47 0.63
Actin
48kDa
1 0.94 1.04 1.41

Figure 4E

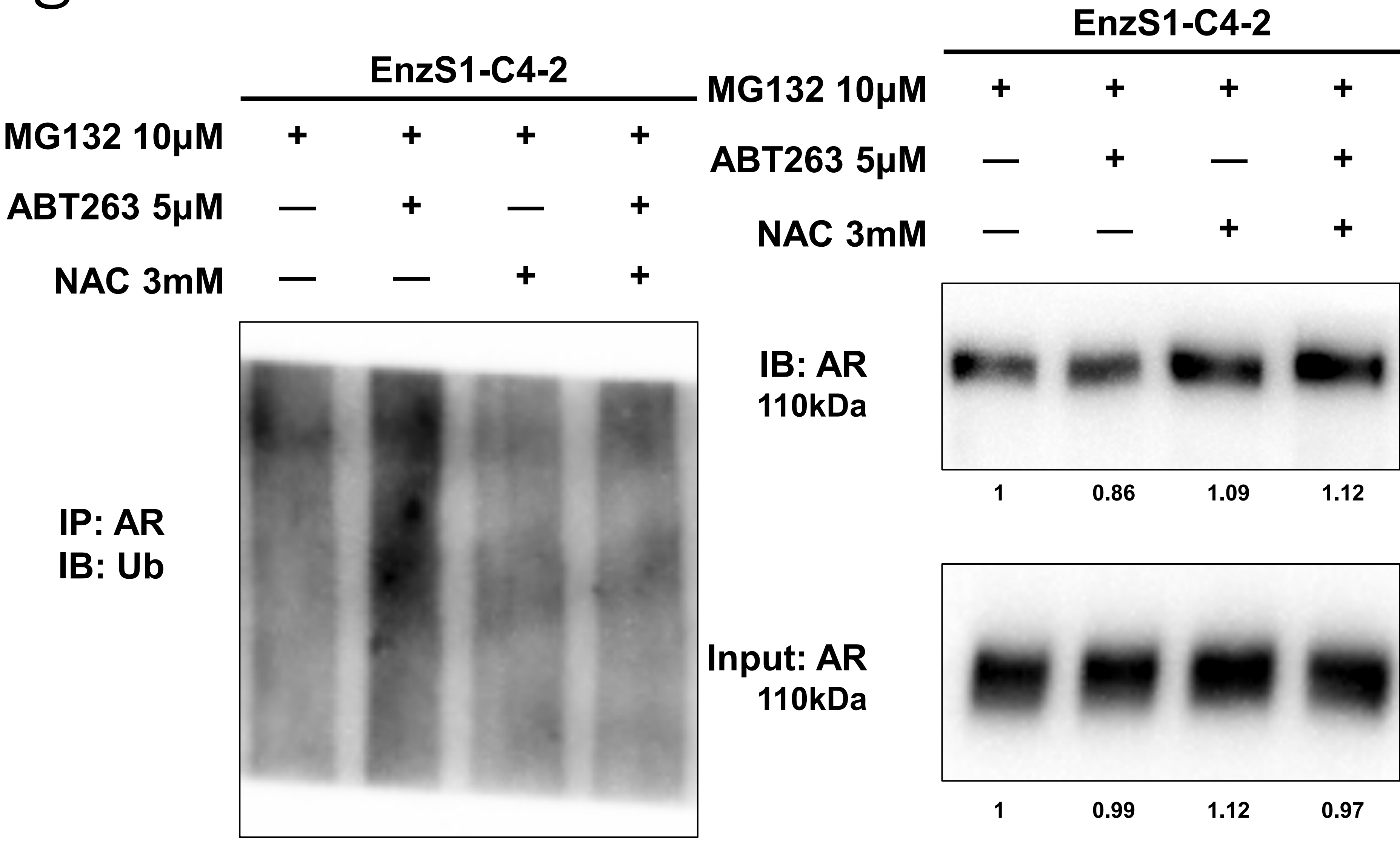
EnzS1-C4-2
MG132 10μM + + + +
ABT263 5μM — + — +
NAC 3mM — — + +
IP: AR
IB: Ub
EnzS1-C4-2
MG132 10μM + + + +
ABT263 5μM — + — +
NAC 3mM — — + +
IB: AR
110kDa
1 0.86 1.09 1.12
Input: AR
110kDa
1 0.99 1.12 0.97

Figure 4F

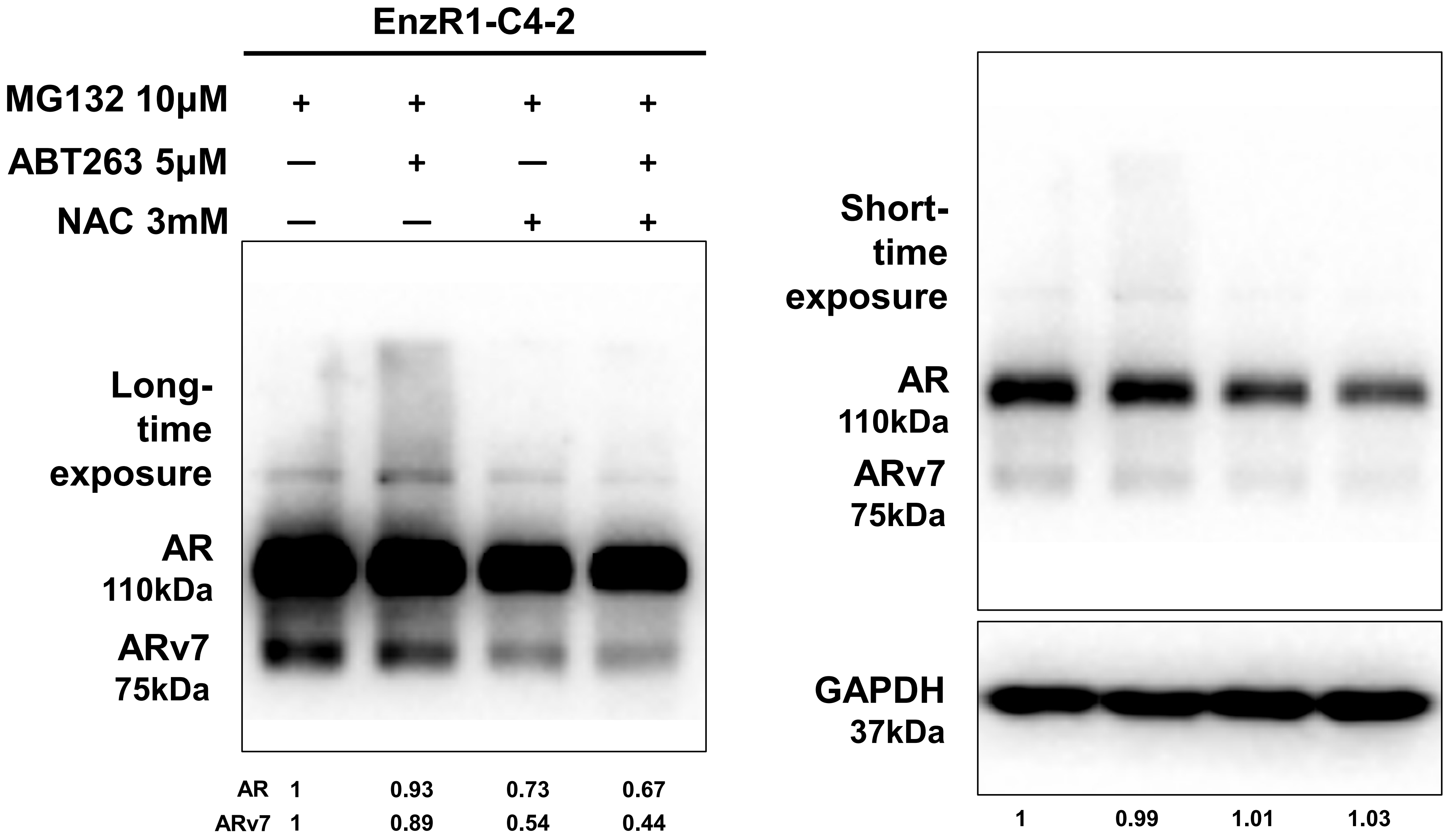
EnzR1-C4-2
MG132 10μM + + + +
ABT263 5μM — + — +
NAC 3mM — — + +
Long-time exposure
AR 110kDa
ARv7 75kDa
AR 1 0.93 0.73 0.67
ARv7 1 0.89 0.54 0.44
Short-time exposure
AR 110kDa
ARv7 75kDa
GAPDH 37kDa
1 0.99 1.01 1.03

Figure 4G

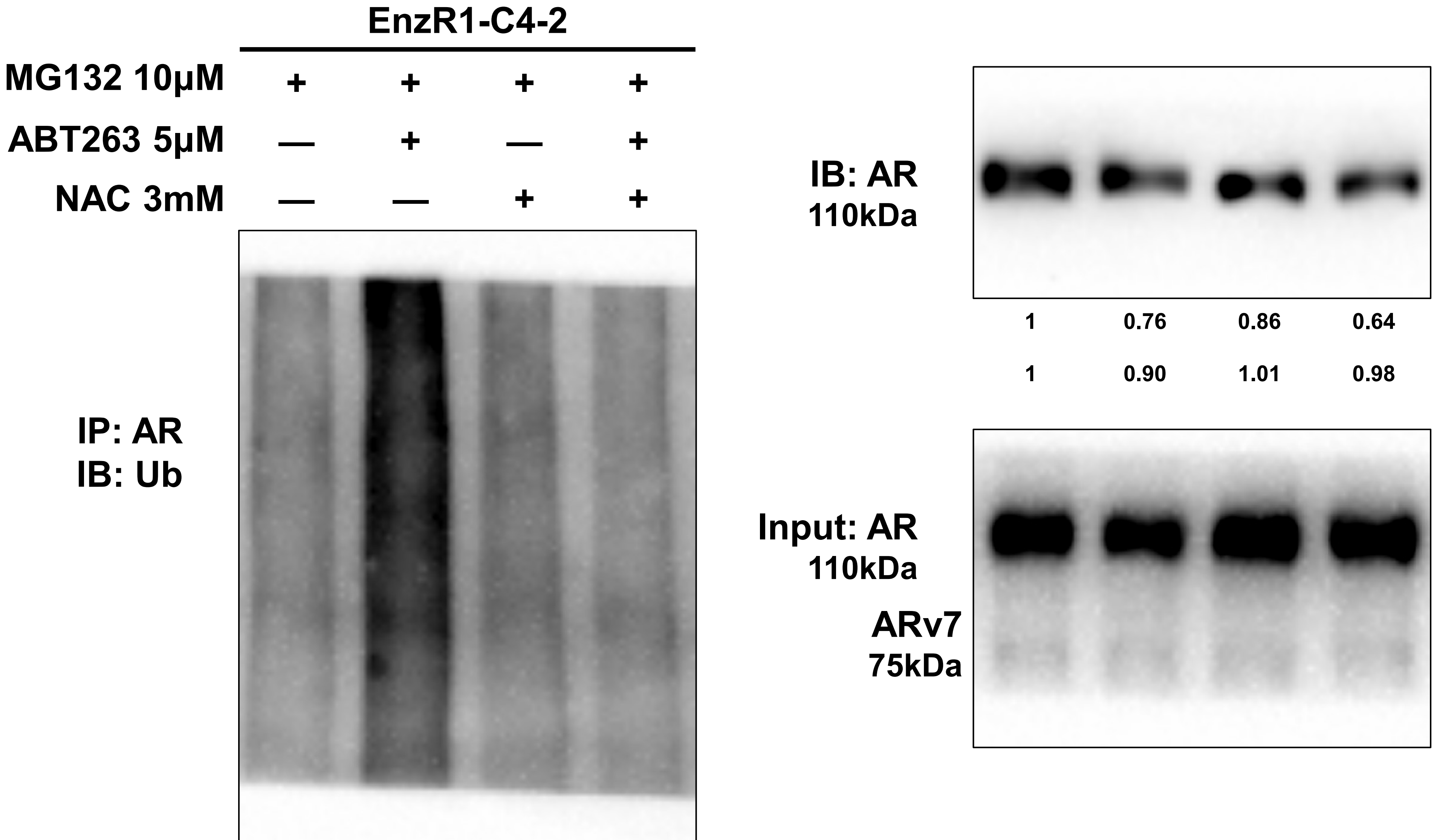

EnzR1-C4-2
MG132 10μM + + + +
ABT263 5μM — + — +
NAC 3mM — — + +
IP: AR
IB: Ub
IB: AR
110kDa
1 0.76 0.86 0.64
1 0.90 1.01 0.98
Input: AR
110kDa
ARv7
75kDa

Figure 5A

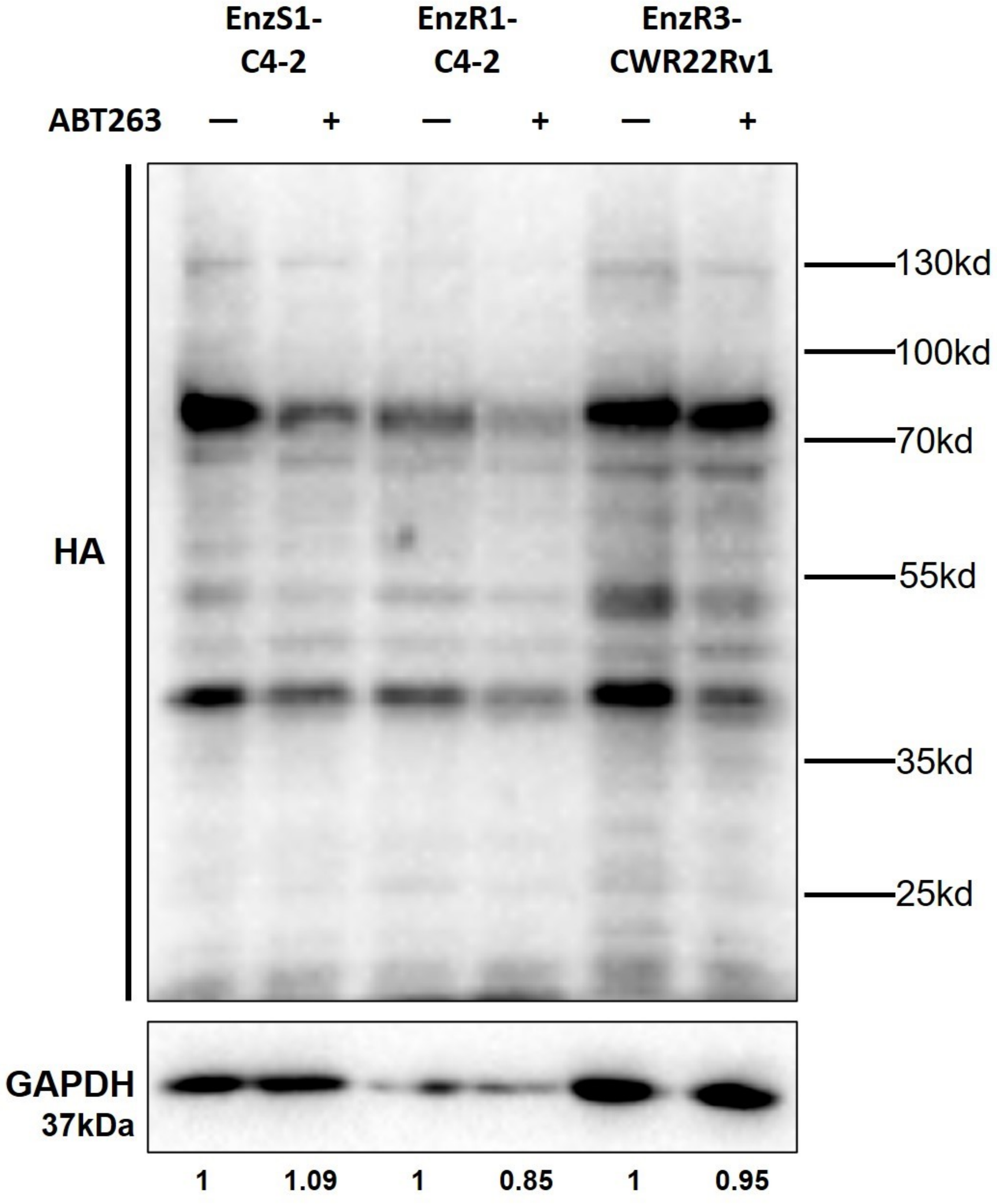

EnzS1-
C4-2
EnzR1-
C4-2
EnzR3-
CWR22Rv1
ABT263
—
+
—
+
—
+
HA
130kd
100kd
70kd
55kd
35kd
25kd
GAPDH
37kDa
1
1.09
1
0.85
1
0.95

Figure 5B

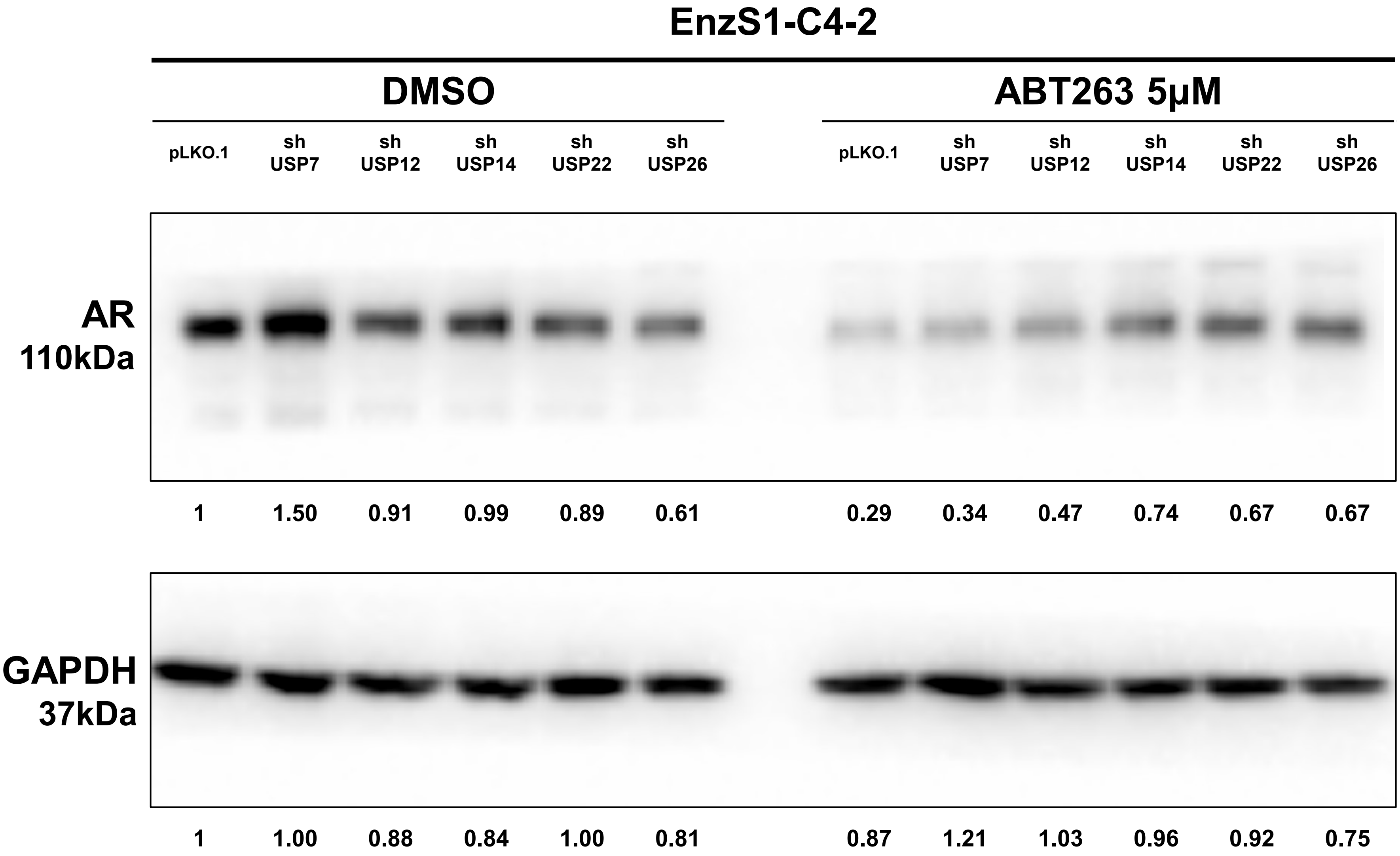
EnzS1-C4-2
DMSO
ABT263 5μM
pLKO.1
sh USP7
sh USP12
sh USP14
sh USP22
sh USP26
pLKO.1
sh USP7
sh USP12
sh USP14
sh USP22
sh USP26
AR 110kDa
1
1.50
0.91
0.99
0.89
0.61
0.29
0.34
0.47
0.74
0.67
0.67
GAPDH 37kDa
1
1.00
0.88
0.84
1.00
0.81
0.87
1.21
1.03
0.96
0.92
0.75

Figure 5C

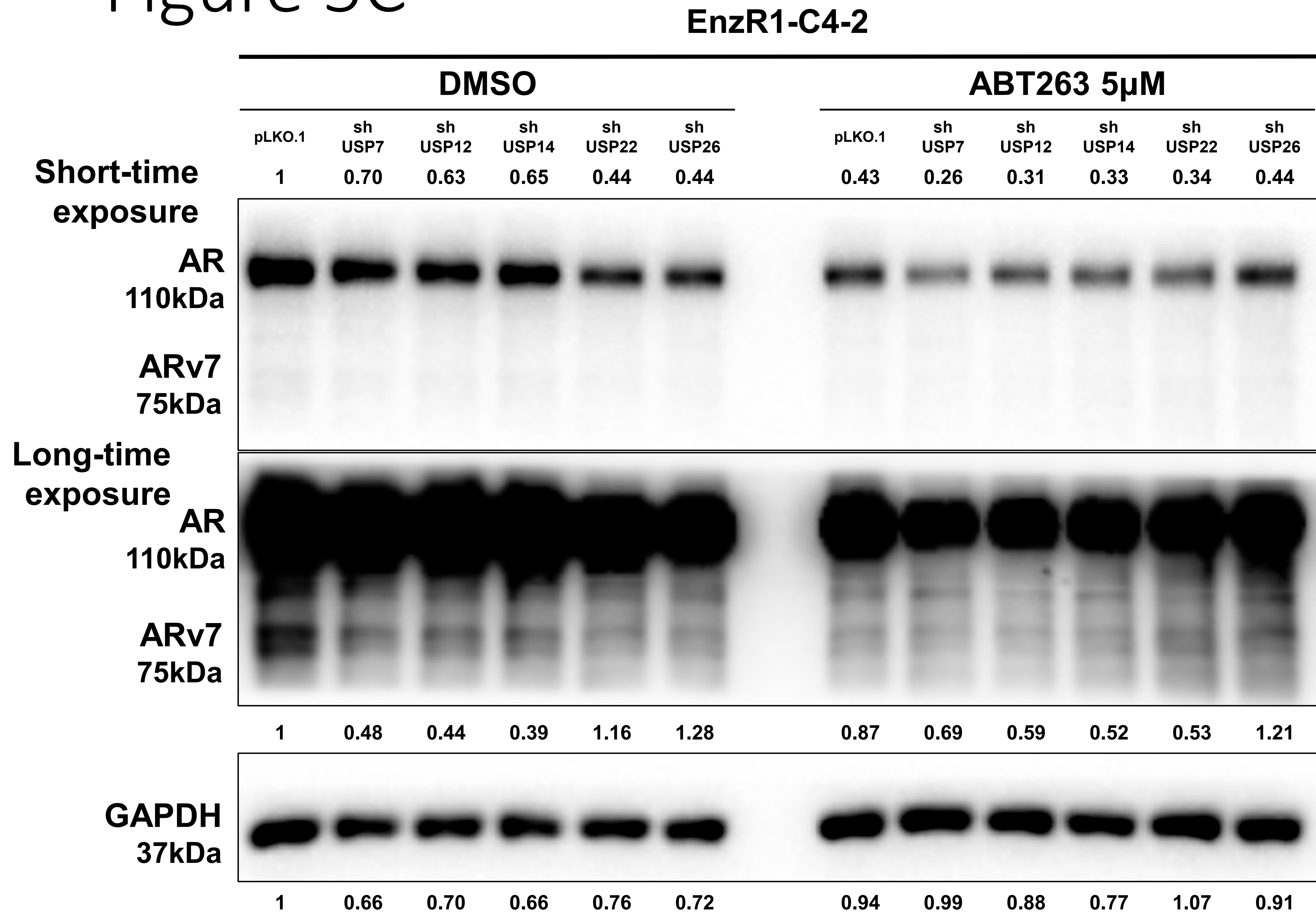

EnzR1-C4-2
DMSO
ABT263 5μM
pLKO.1
sh USP7
sh USP12
sh USP14
sh USP22
sh USP26
pLKO.1
sh USP7
sh USP12
sh USP14
sh USP22
sh USP26
Short-time exposure
1 0.70 0.63 0.65 0.44 0.44 0.43 0.26 0.31 0.33 0.34 0.44
AR 110kDa
ARv7 75kDa
Long-time exposure
AR 110kDa
ARv7 75kDa
1 0.48 0.44 0.39 1.16 1.28 0.87 0.69 0.59 0.52 0.53 1.21
GAPDH 37kDa
1 0.66 0.70 0.66 0.76 0.72 0.94 0.99 0.88 0.77 1.07 0.91

Figure 5D

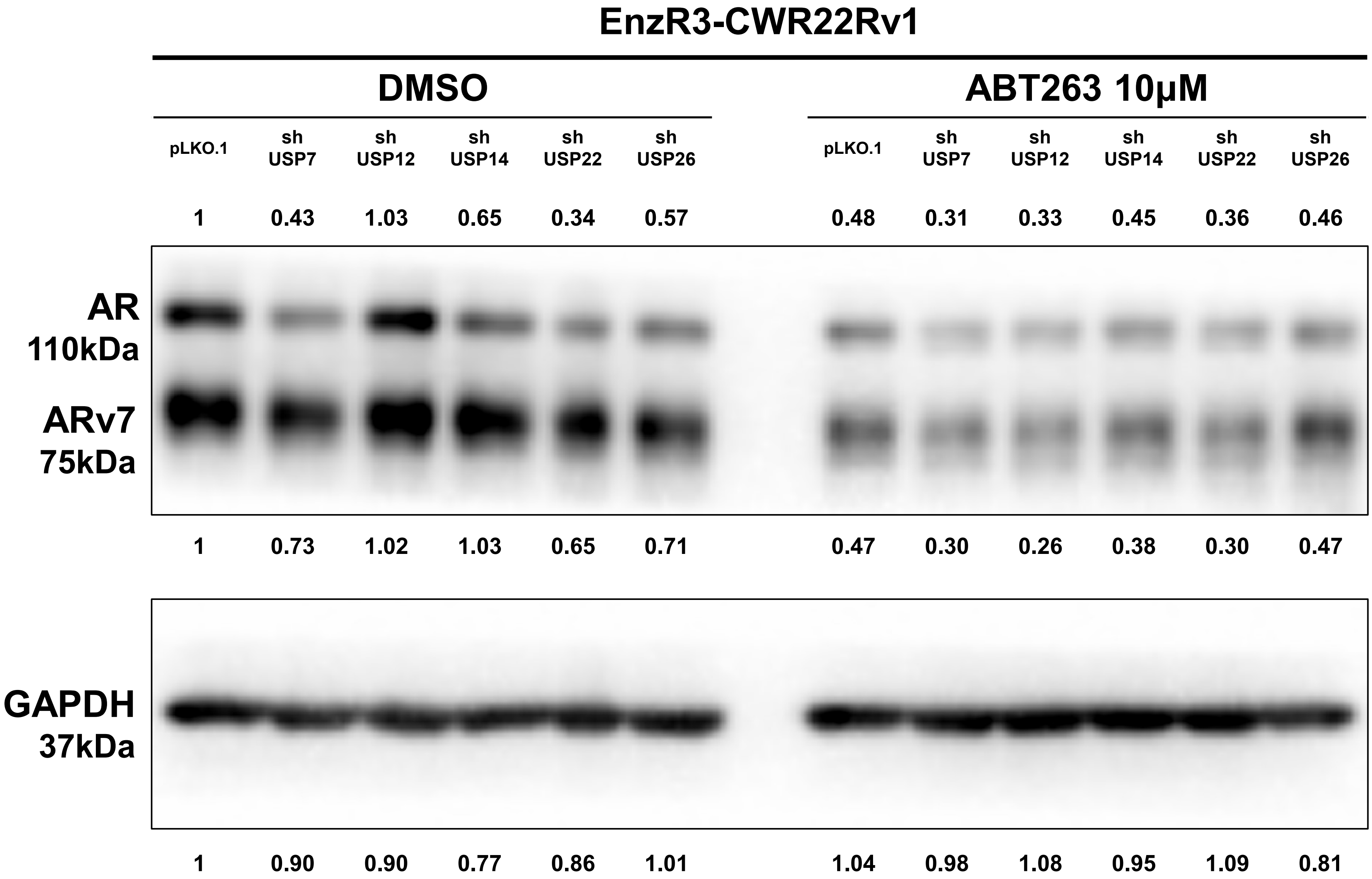
EnzR3-CWR22Rv1
DMSO
ABT263 10μM
pLKO.1
sh USP7
sh USP12
sh USP14
sh USP22
sh USP26
pLKO.1
sh USP7
sh USP12
sh USP14
sh USP22
sh USP26
1 0.43 1.03 0.65 0.34 0.57 0.48 0.31 0.33 0.45 0.36 0.46
AR 110kDa
ARv7 75kDa
1 0.73 1.02 1.03 0.65 0.71 0.47 0.30 0.26 0.38 0.30 0.47
GAPDH 37kDa
1 0.90 0.90 0.77 0.86 1.01 1.04 0.98 1.08 0.95 1.09 0.81

Figure 5E

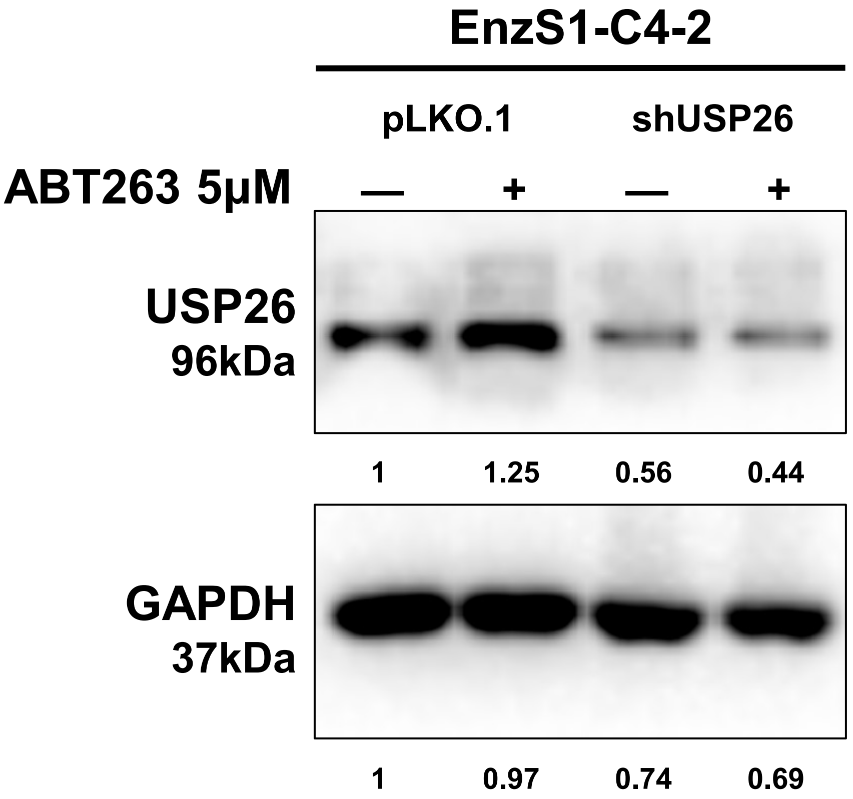

EnzS1-C4-2
pLKO.1
shUSP26
ABT263 5μM
—
+
—
+
USP26
96kDa
1
1.25
0.56
0.44
GAPDH
37kDa
1
0.97
0.74
0.69

Figure 5F

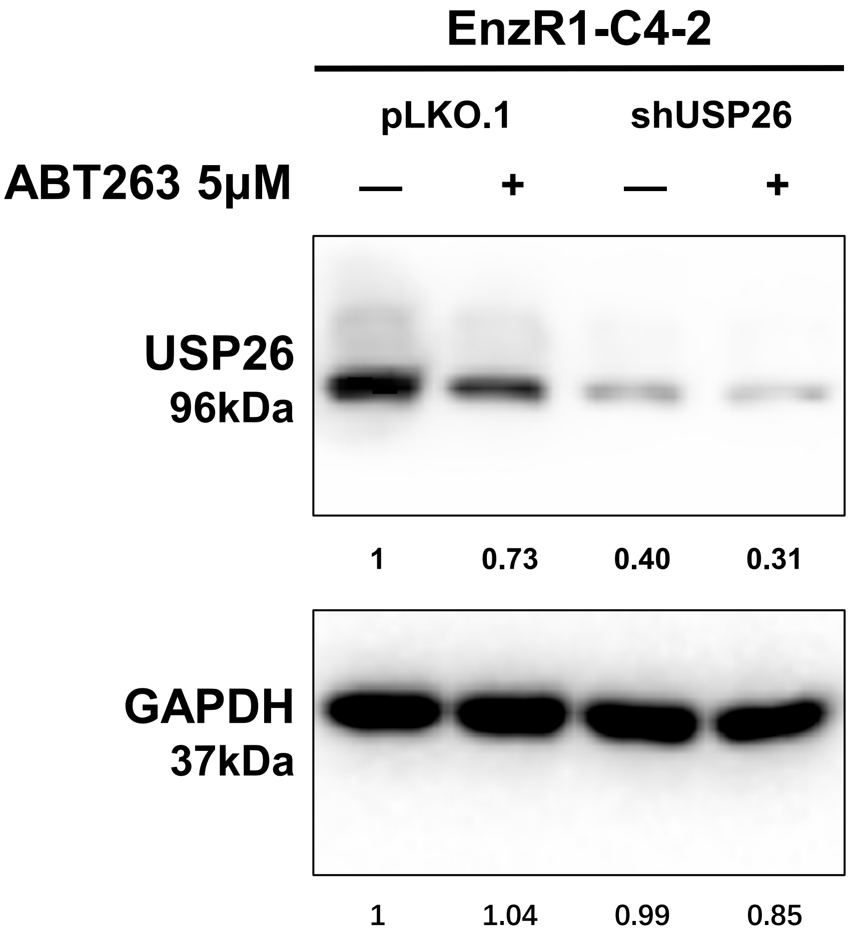
EnzR1-C4-2
pLKO.1
shUSP26
ABT263 5μM
—
+
—
+
USP26
96kDa
1
0.73
0.40
0.31
GAPDH
37kDa
1
1.04
0.99
0.85

Figure 5G

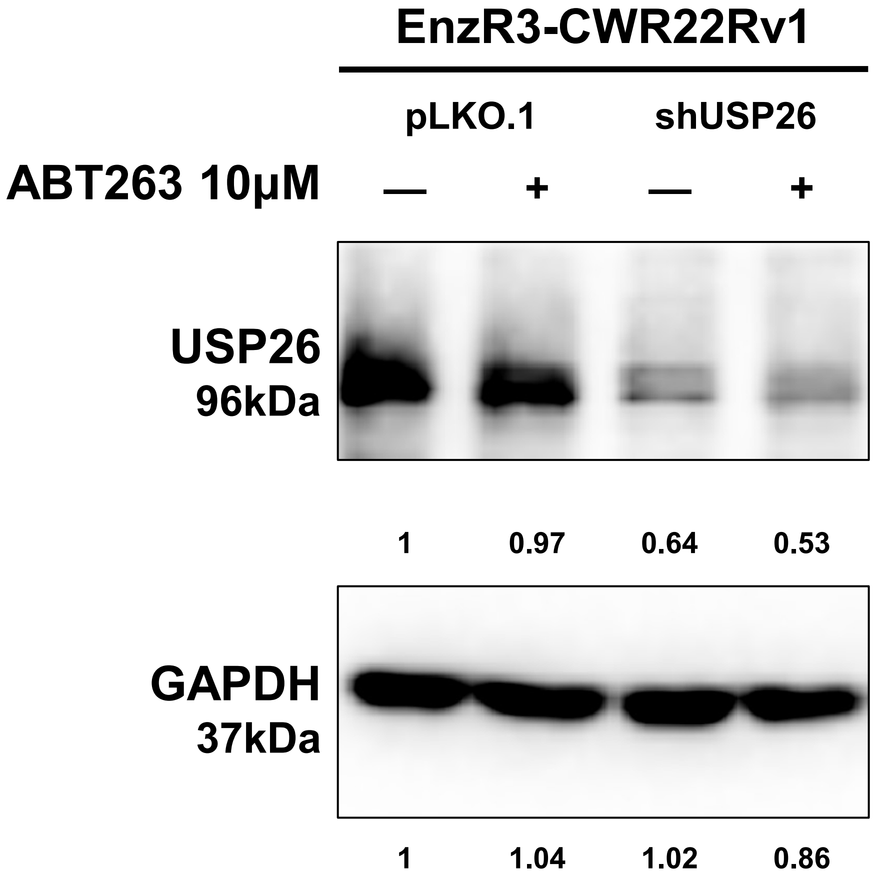
EnzR3-CWR22Rv1
pLKO.1
shUSP26
ABT263 10μM
—
+
—
+
USP26
96kDa
1
0.97
0.64
0.53
GAPDH
37kDa
1
1.04
1.02
0.86

Figure 6A

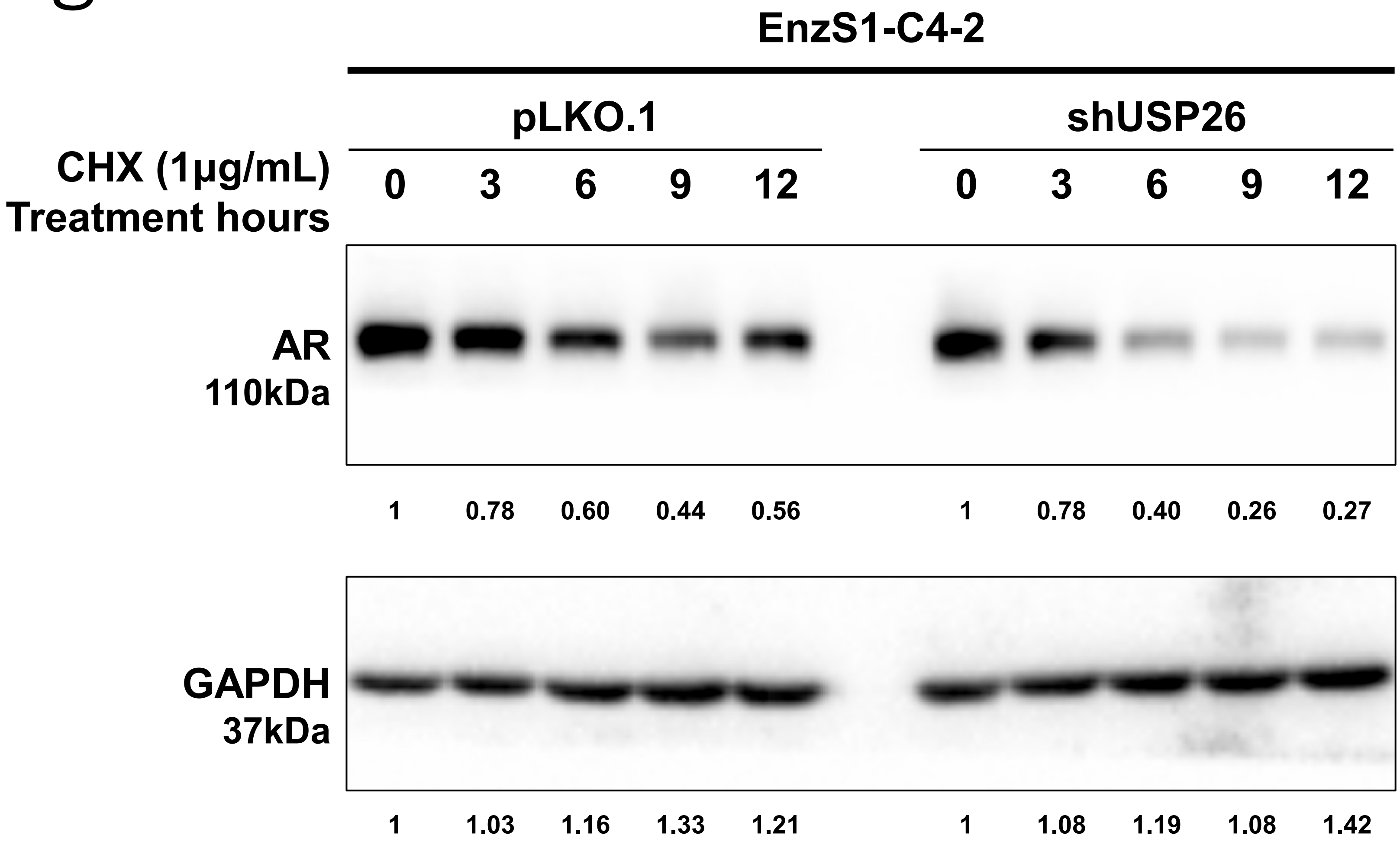
EnzS1-C4-2
pLKO.1
shUSP26
CHX (1μg/mL)
Treatment hours
0 3 6 9 12
0 3 6 9 12
AR
110kDa
1 0.78 0.60 0.44 0.56
1 0.78 0.40 0.26 0.27
GAPDH
37kDa
1 1.03 1.16 1.33 1.21
1 1.08 1.19 1.08 1.42

Figure 6B

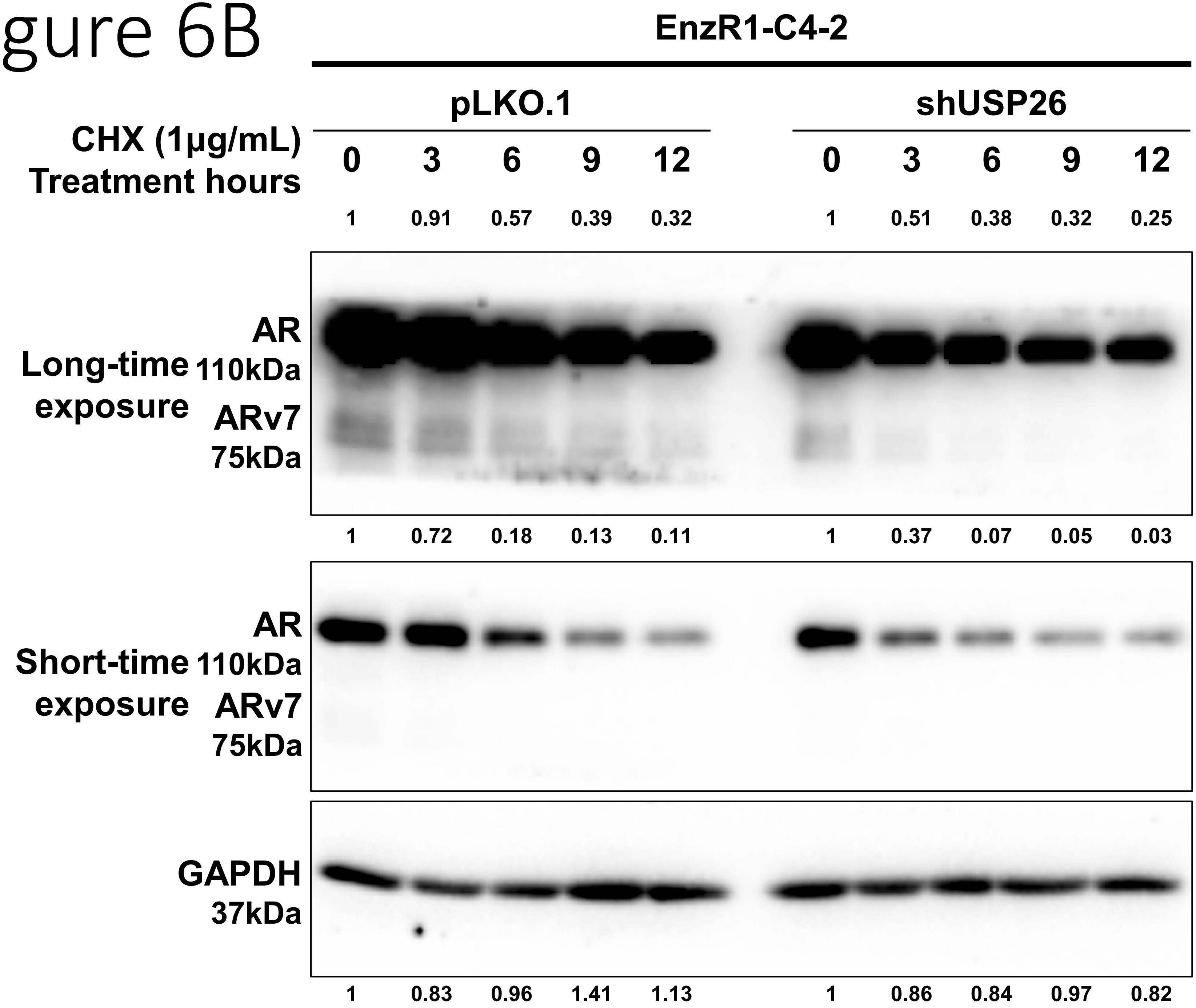
EnzR1-C4-2
pLKO.1
shUSP26
CHX (1μg/mL)
Treatment hours
0 3 6 9 12
0 3 6 9 12
1 0.91 0.57 0.39 0.32
1 0.51 0.38 0.32 0.25
AR
110kDa
ARv7
75kDa
Long-time exposure
1 0.72 0.18 0.13 0.11
1 0.37 0.07 0.05 0.03
AR
110kDa
ARv7
75kDa
Short-time exposure
GAPDH
37kDa
1 0.83 0.96 1.41 1.13
1 0.86 0.84 0.97 0.82

Figure 6C

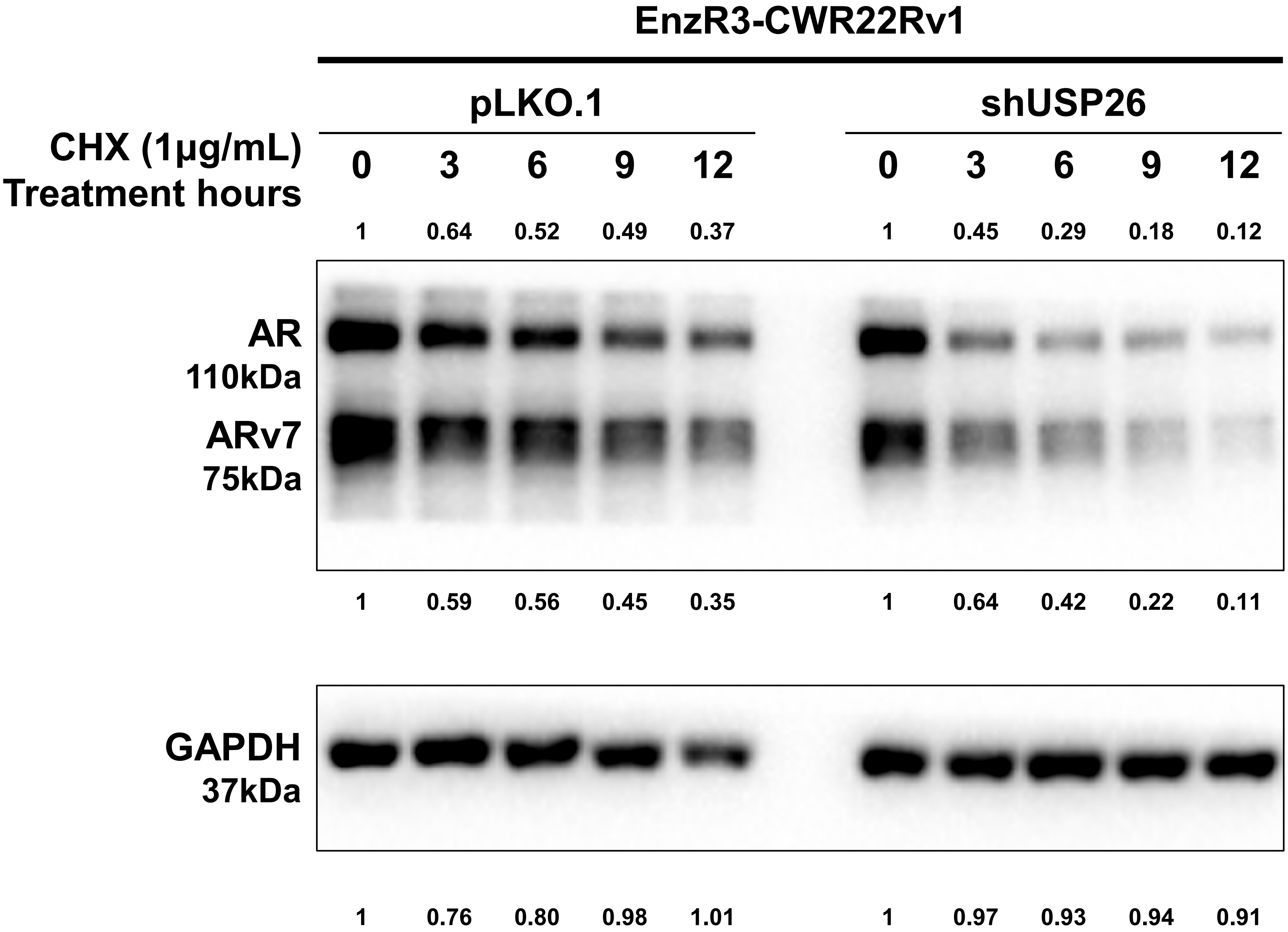
EnzR3-CWR22Rv1
pLKO.1
shUSP26
CHX (1µg/mL)
Treatment hours
0 3 6 9 12
0 3 6 9 12
1 0.64 0.52 0.49 0.37
1 0.45 0.29 0.18 0.12
AR
110kDa
ARv7
75kDa
1 0.59 0.56 0.45 0.35
1 0.64 0.42 0.22 0.11
GAPDH
37kDa
1 0.76 0.80 0.98 1.01
1 0.97 0.93 0.94 0.91

Figure 6D

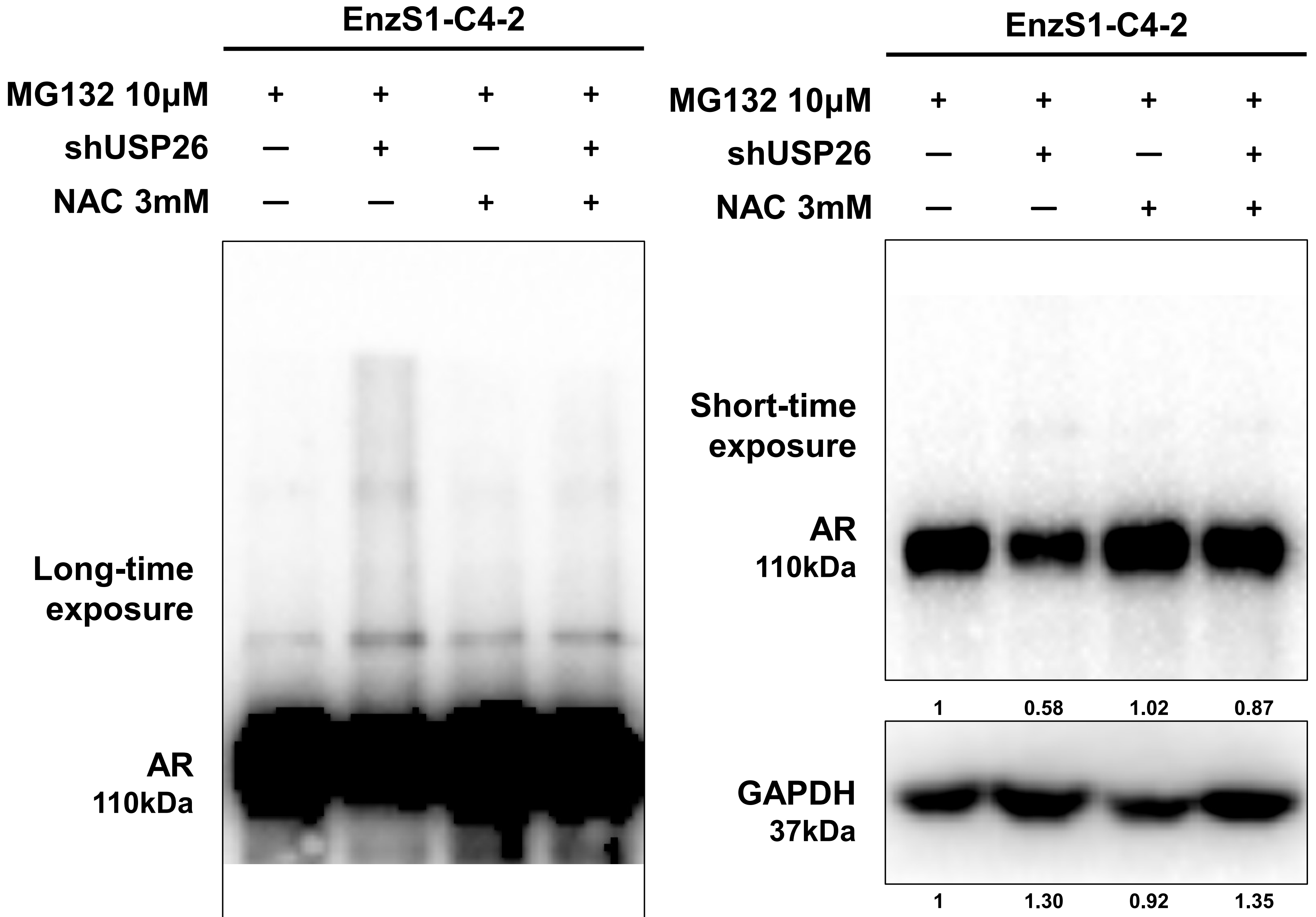
EnzS1-C4-2
MG132 10μM + + + +
shUSP26 — + — +
NAC 3mM — — + +
Long-time exposure
AR
110kDa
EnzS1-C4-2
MG132 10μM + + + +
shUSP26 — + — +
NAC 3mM — — + +
Short-time exposure
AR
110kDa
1 0.58 1.02 0.87
GAPDH
37kDa
1 1.30 0.92 1.35

Figure 6E

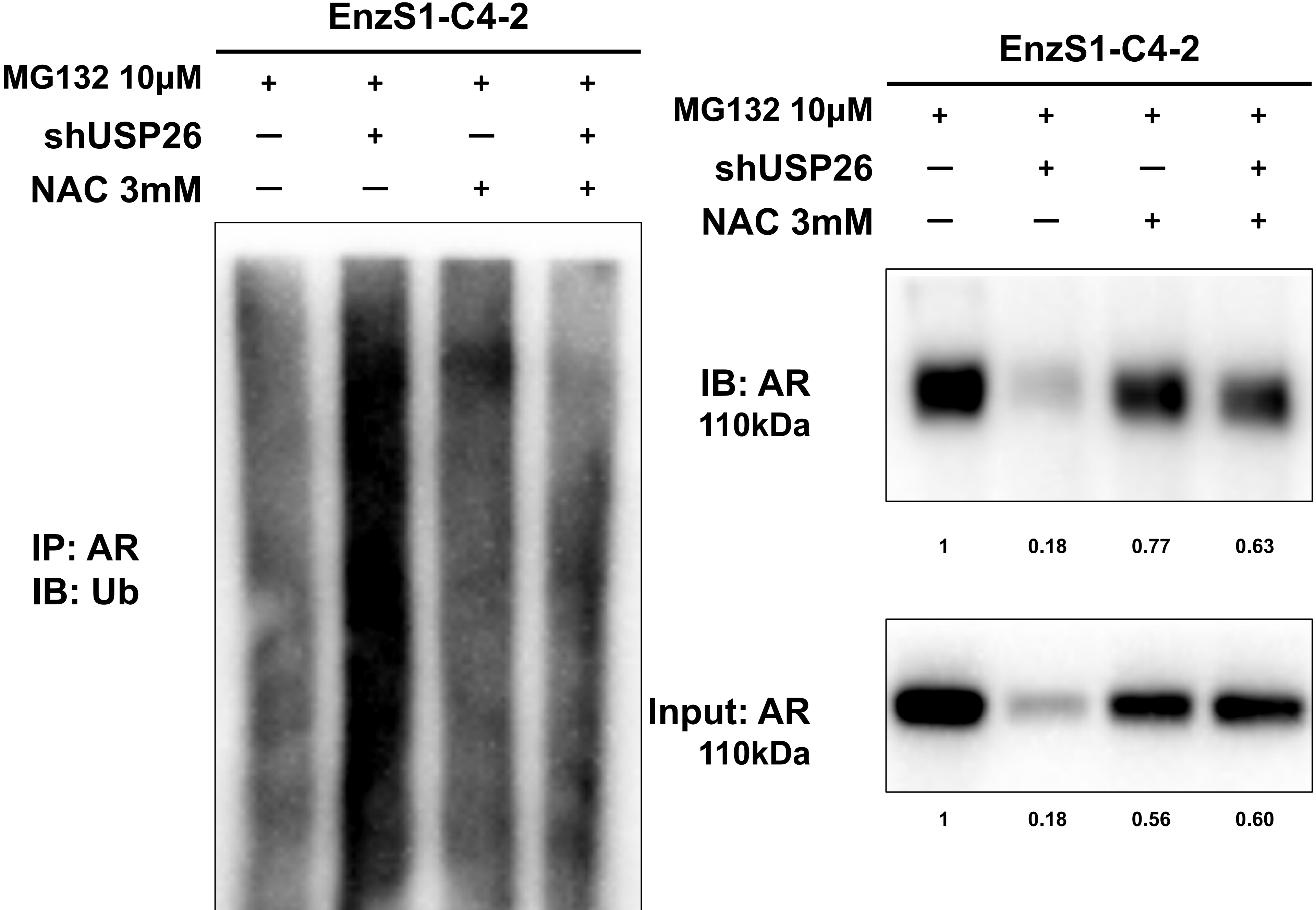
EnzS1-C4-2
MG132 10μM + + + +
shUSP26 — + — +
NAC 3mM — — + +
IP: AR
IB: Ub
EnzS1-C4-2
MG132 10μM + + + +
shUSP26 — + — +
NAC 3mM — — + +
IB: AR
110kDa
1 0.18 0.77 0.63
Input: AR
110kDa
1 0.18 0.56 0.60

Figure 6F

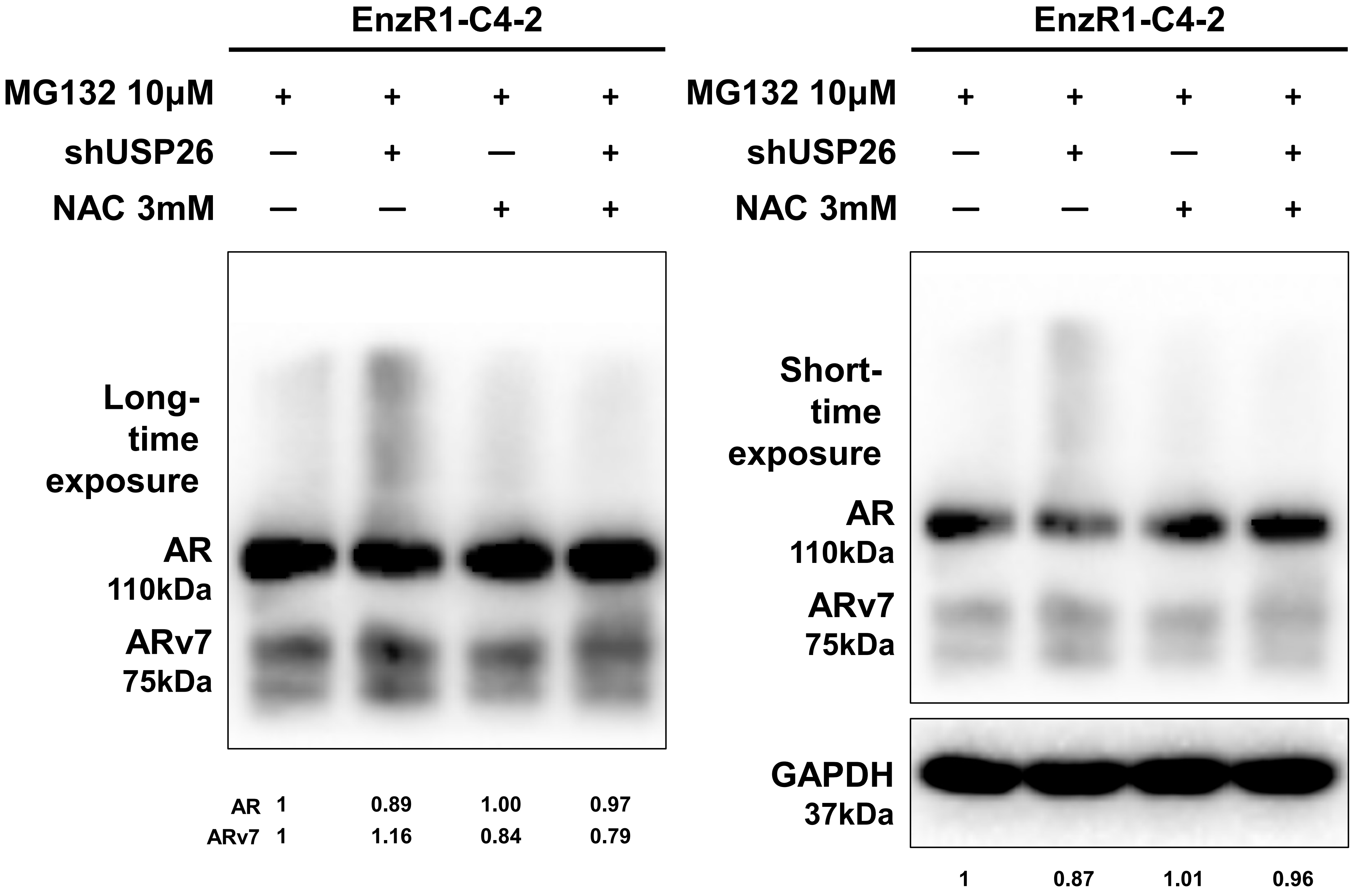
EnzR1-C4-2
MG132 10μM + + + +
shUSP26 — + — +
NAC 3mM — — + +
Long-time exposure
AR 110kDa
ARv7 75kDa
AR 1 0.89 1.00 0.97
ARv7 1 1.16 0.84 0.79
EnzR1-C4-2
MG132 10μM + + + +
shUSP26 — + — +
NAC 3mM — — + +
Short-time exposure
AR 110kDa
ARv7 75kDa
GAPDH 37kDa
1 0.87 1.01 0.96

Figure 6G

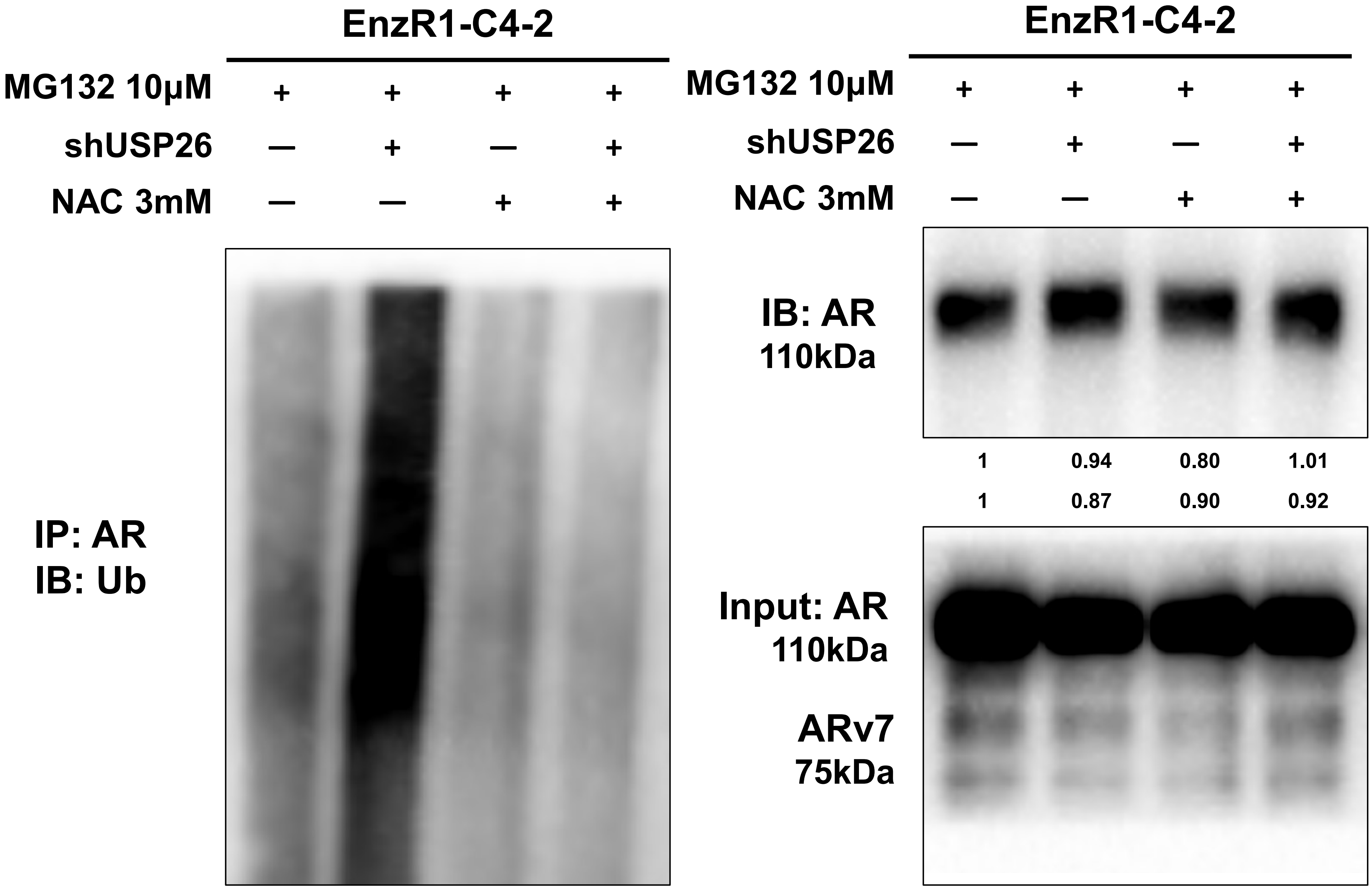
EnzR1-C4-2
MG132 10μM + + + +
shUSP26 — + — +
NAC 3mM — — + +
IP: AR
IB: Ub
EnzR1-C4-2
MG132 10μM + + + +
shUSP26 — + — +
NAC 3mM — — + +
IB: AR
110kDa
1 0.94 0.80 1.01
1 0.87 0.90 0.92
Input: AR
110kDa
ARv7
75kDa

Figure 6H

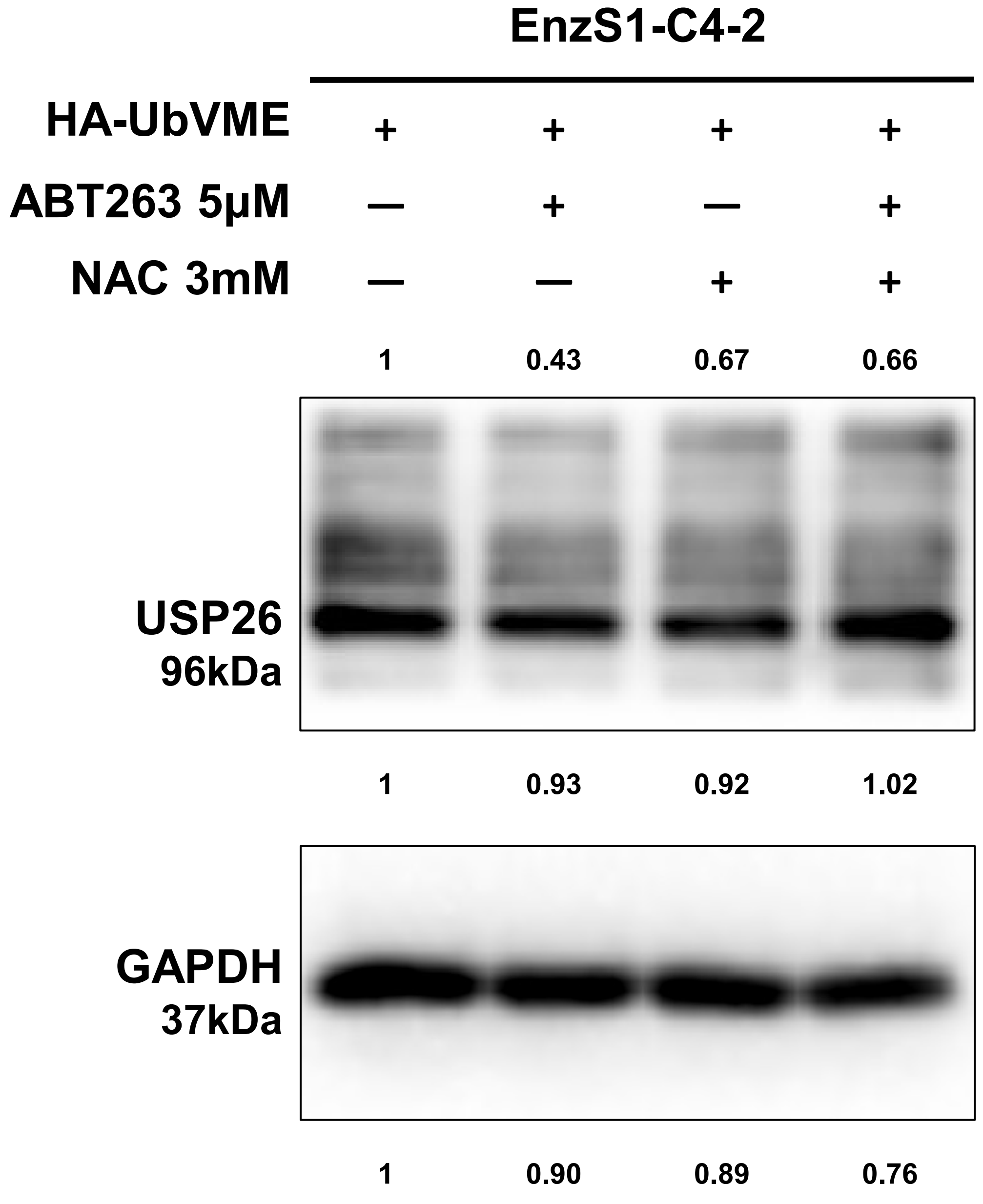
EnzS1-C4-2
HA-UbVME + + + +
ABT263 5μM — + — +
NAC 3mM — — + +
1 0.43 0.67 0.66
USP26
96kDa
1 0.93 0.92 1.02
GAPDH
37kDa
1 0.90 0.89 0.76

Figure 6I

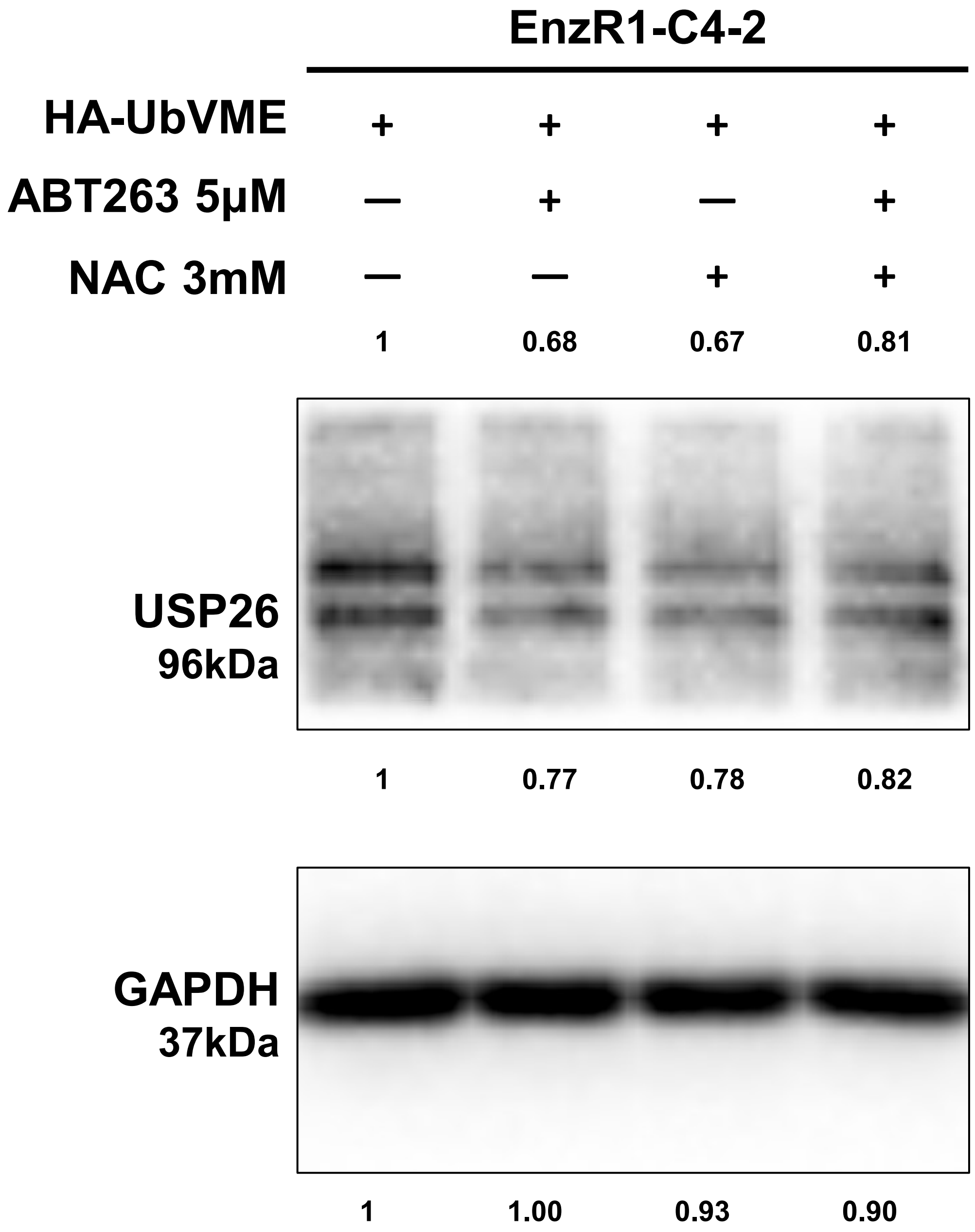
EnzR1-C4-2
HA-UbVME + + + +
ABT263 5μM — + — +
NAC 3mM — — + +
1 0.68 0.67 0.81
USP26
96kDa
1 0.77 0.78 0.82
GAPDH
37kDa
1 1.00 0.93 0.90

Figure 6J

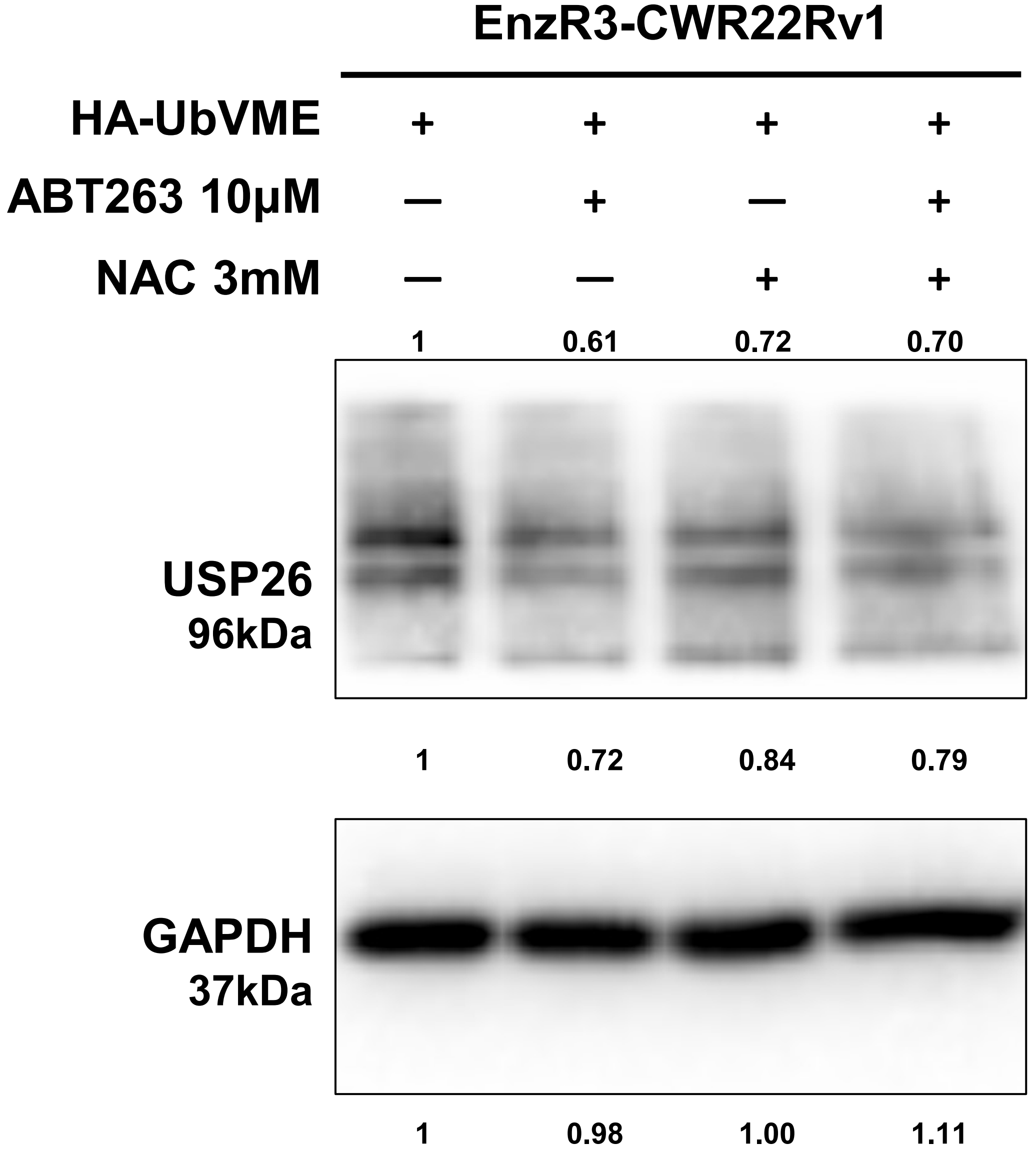
EnzR3-CWR22Rv1
HA-UbVME + + + +
ABT263 10μM — + — +
NAC 3mM — — + +
1 0.61 0.72 0.70
USP26
96kDa
1 0.72 0.84 0.79
GAPDH
37kDa
1 0.98 1.00 1.11
