# Supplementary material for: Preclinical Study Using ABT263 to Increase Enzalutamide Sensitivity to Suppress Prostate Cancer Progression Via Targeting BCL2/ROS/USP26 Axis Through Altering ARv7 Protein Degradation
Source: Cancers (Basel). 2020 Mar 30;12(4):831. doi: 10.3390/cancers12040831 (PMC7226306; doi:10.3390/cancers12040831)
Supplement: Supplementary file 1 [file cancers-12-00831-s001.zip › cancers-732702 supplementary/Supplement figures and tables.pdf]

cancers

MDPI

Article

Preclinical Study Using ABT263 to Increase Enzalutamide Sensitivity to Suppress Prostate Cancer Progression Via Targeting BCL2/ROS/USP26 Axis Through Altering ARv7 Protein Degradation

Hua Xu [1,2,3,4#], Yin Sun [3,4#], Chi-Ping Huang [5], Bosen You [3,4], Dingwei Ye [1,2,*] and Chawnshang Chang [3,4,5*]

[1] Department of Urology, Fudan University Shanghai Cancer Center, Shanghai 200032, China
[2] Department of Oncology, Shanghai Medical College, Fudan University, Shanghai 200032, China
[3] George Whipple Lab for Cancer Research, Departments of Pathology, Urology, and Radiation Oncology, University of Rochester Medical Center, Rochester, NY 14646, USA
[4] The Wilmot Cancer Institute, University of Rochester Medical Center, Rochester, NY 14646, USA
[5] Sex Hormone Research Center and Department of Urology, China Medical University, Taichung 404, Taiwan
# These authors contributed equally to this work.
* Correspondence: dwyeli@163.com (D.Y.); chang@urmc.rochester.edu (C.C.)

Supplementary Figures:

Supplement Fig. 1

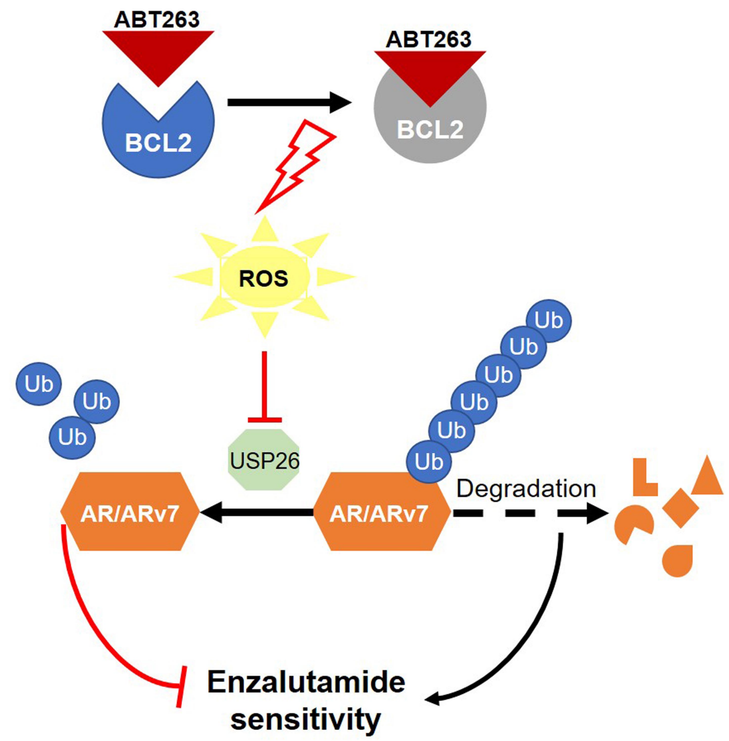

Supplement Fig. 1. Model of ABT263 increased Enz sensitivity of PCa. ABT263, as a selective antagonist of BCL2, induces cellular ROS and inhibits USP26 activity. The ubiquitination of AR/ARv7 increases due to inhibition of USP26, resulting in increased degradation of AR/ARv7. Decreased AR/ARv7 expression by ABT263 contributes to increased Enz sensitivity of PCa.

Supplement Fig. 2

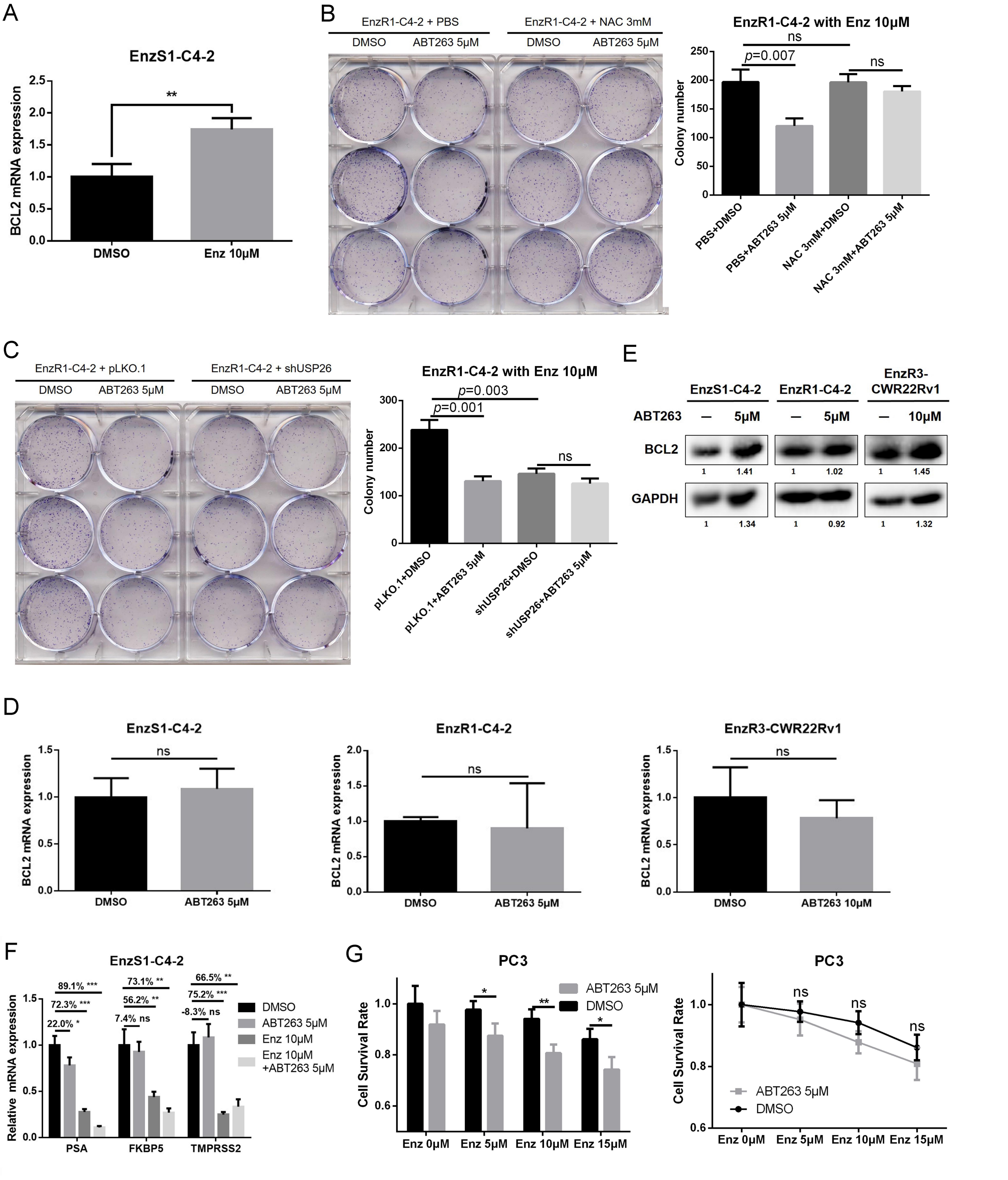

Supplement Fig. 2. A: BCL2 mRNA expression increased in response to Enz treatment for 48 hours in EnzS1-C4-2 cells. **B-C**: ABT263 suppresses EnzR1-C4-2 cell colony formation, which could be reversed by NAC (**B**) and shUSP26 (**C**). **D-E**: ABT263 doesn't decrease BCL2 expression. BCL2 mRNA and protein expression were detected after 48 hours ABT263 treatment. Q-PCR assay was applied to measure BCL2 mRNA (**D**), and western blot assay was applied to measure BCL2 protein expression (**E**) in EnzS1-C4-2 cells, EnzR1-C4-2 cells, and EnzR3-CWR22Rv1 cells. **F**: Combination of ABT263 and Enz treatment better suppresses AR target gene expression. Q-PCR assay was applied to measure the mRNA expression of AR target genes (PSA, FKBP5, and TMPRSS2) in EnzS1-C4-2 cells after combination treatment of ABT263 and Enz or ABT263 and Enz treatment alone. **G**: Targeting BCL2 with ABT263 doesn't increase sensitivity for Enzalutamide in PC3 cells. Data are presented as Mean ± SD. *: $p < 0.05$, **: $p < 0.01$, ***: $p < 0.001$, ns: not significant.

Supplement Fig. 3

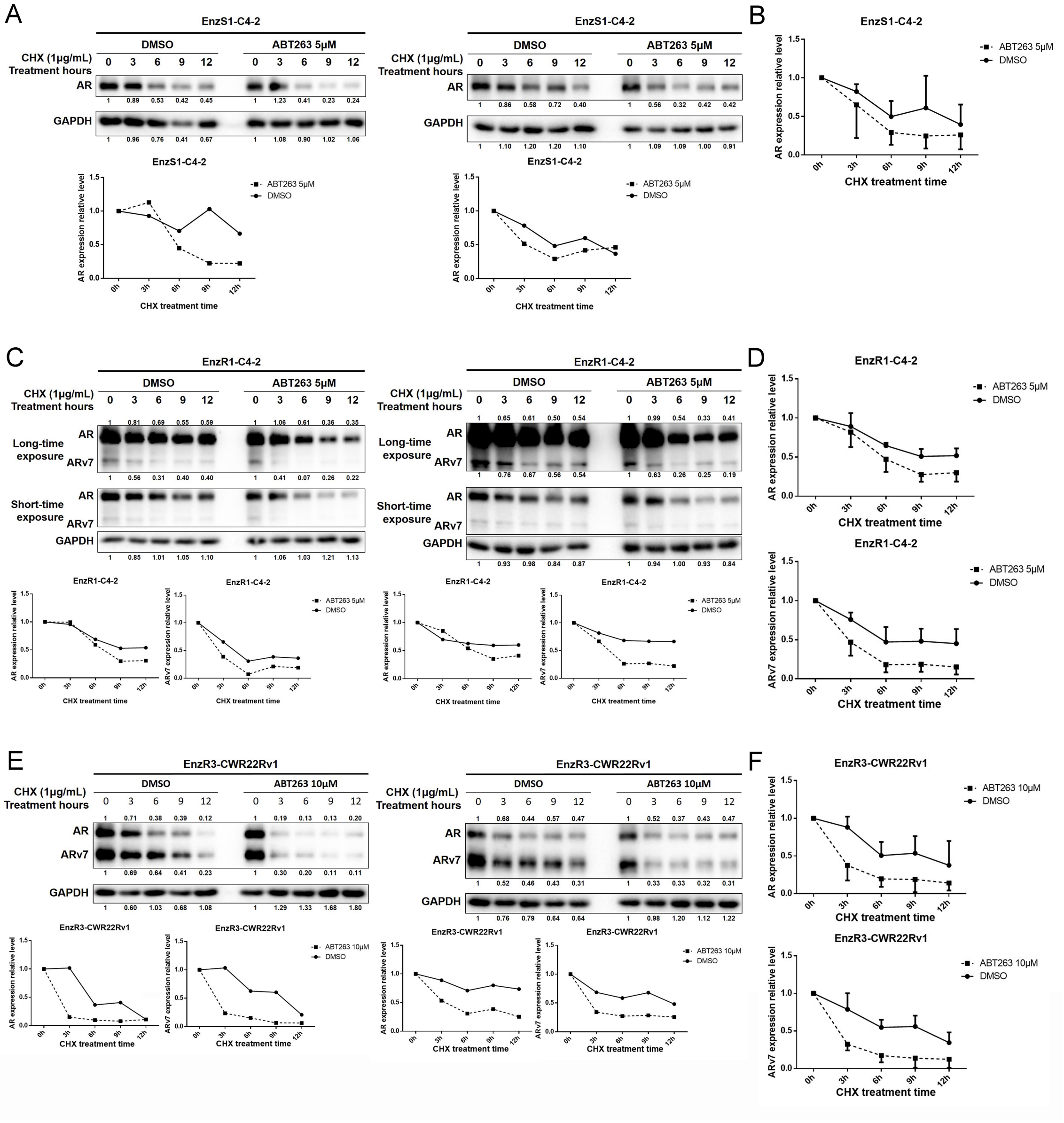

Supplement Fig. 3. ABT263 decreases AR and ARv7 protein stability. As described in **Fig. 2**, stability of AR and ARv7 in EnzS1-C4-2 cells (**A**), in EnzR1-C4-2 cells (**C**) and in EnzR3-CWR22Rv1 cells (**E**) was measured by western blot assay another two times. Chemiluminescence on the western blot detected with short and long length of time, are shown in **C** and **E**. Note that ARv7 is more visible with longer exposure time. Together with data in **Fig. 2C-E**, line graphs of AR/ARv7 protein stability were drawn, showing similar decreasing trend of AR/ARv7 stability in EnzS1-C4-2 (**B**), EnzR1-C4-2 (**D**), and EnzR3-CWR22Rv1 (**F**) cells after ABT263 treatment. Data are presented as Mean ± SD.

Supplement Fig. 4

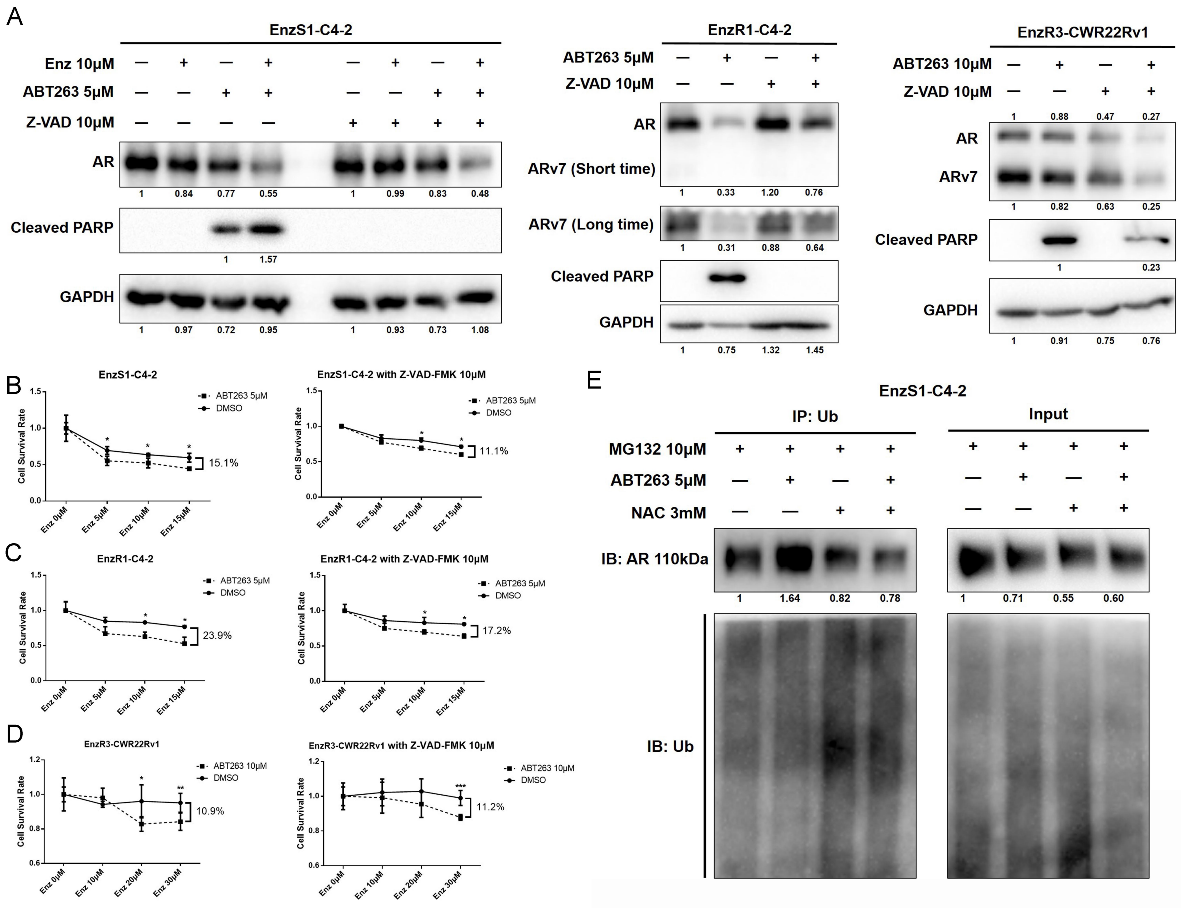

Supplement Fig. 4. ABT263-induced apoptosis didn't contribute to the increase of Enz-sensitivity and AR/ARv7 degradation. **A**: ABT263-induced apoptosis didn't contribute to AR and ARv7 degradation. Z-VAD-FMK (Z-VAD) was used to reverse the cell apoptosis induced by ABT263. Western blot assay was applied to measure AR and ARv7 protein expression in EnzS1-C4-2 cells, EnzR1-C4-2 cells, and EnzR3-CWR22Rv1 cells. Two exposures, short and long time, are shown in EnzR1-C4-2 cells. Note that ARv7 in EnzR1-C4-2 cells is more visible with longer exposure time. **B-D**: ABT263-induced apoptosis didn't contribute to the increase of Enz-sensitivity. MTT proliferation assays were used to detect cell proliferation rates in EnzS1-C4-2 cells (**B**), EnzR1-C4-2 cells (**C**), and EnzR3-CWR22Rv1 cells (**D**). **E**: NAC reverses the ABT263 increased ubiquitination of AR in EnzS1-C4-2. Data are presented as Mean ± SD. *: $p < 0.05$, **: $p < 0.01$, ***: $p < 0.001$.

Supplement Fig. 5

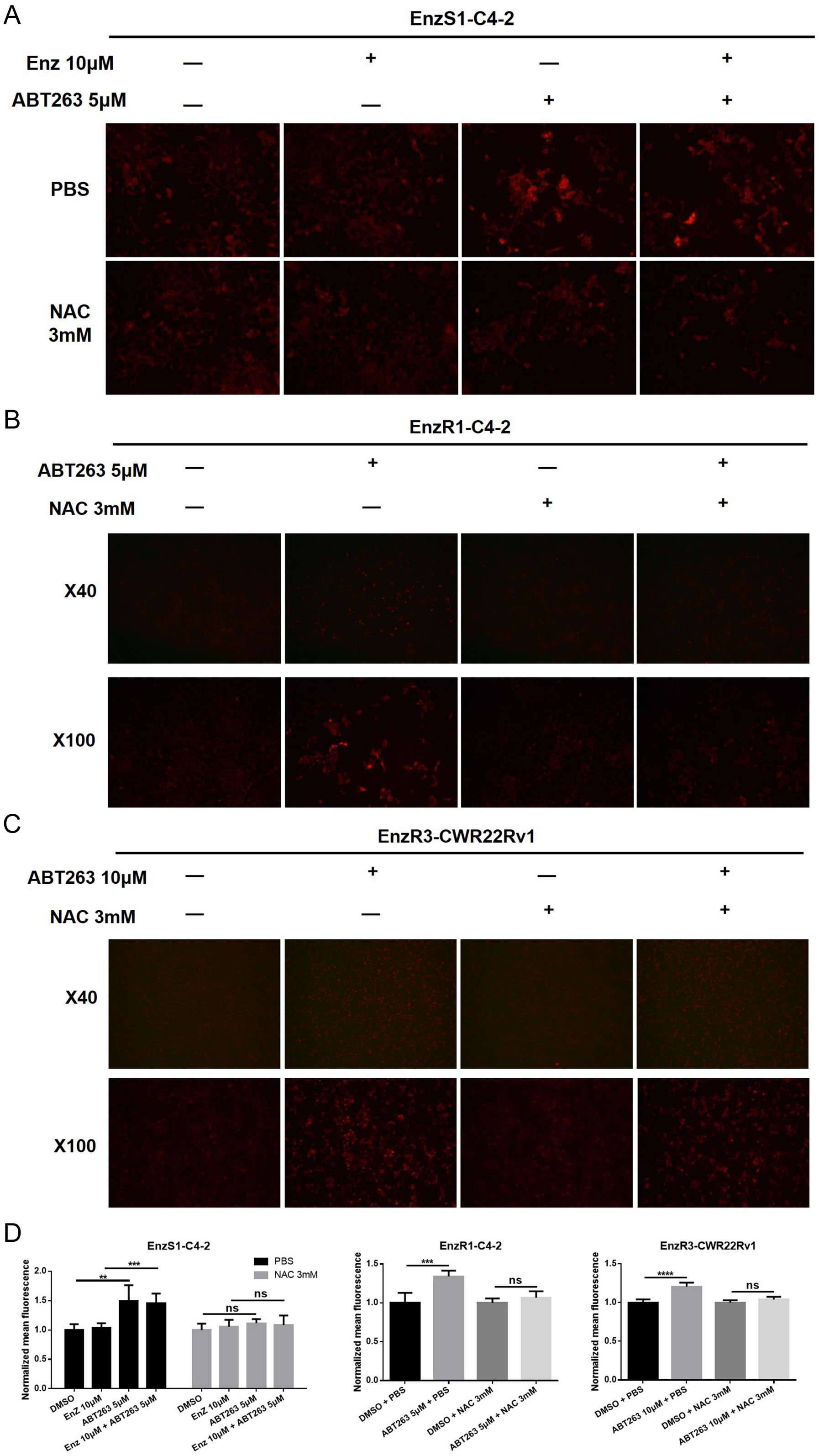

Supplement Fig. 5. NAC reverses cellular ROS induced by ABT263. **A-D**: DHE staining was applied to detect cellular ROS level in EnzS1-C4-2 cells (**A**), EnzR1-C4-2 cells (**B**) and EnzR3-CWR22Rv1 cells (**C**). Fluorescence (excitation wavelength 485 nm and an emission wavelength 580 nm) was measured to quantify the cellular ROS level and showed that NAC reversed cellular ROS induced by ABT263 (**D**). Data are presented as Mean ± SD. **: $p < 0.01$, ***: $p < 0.001$, ****: $p < 0.0001$, ns: not significant.

Supplement Fig. 6

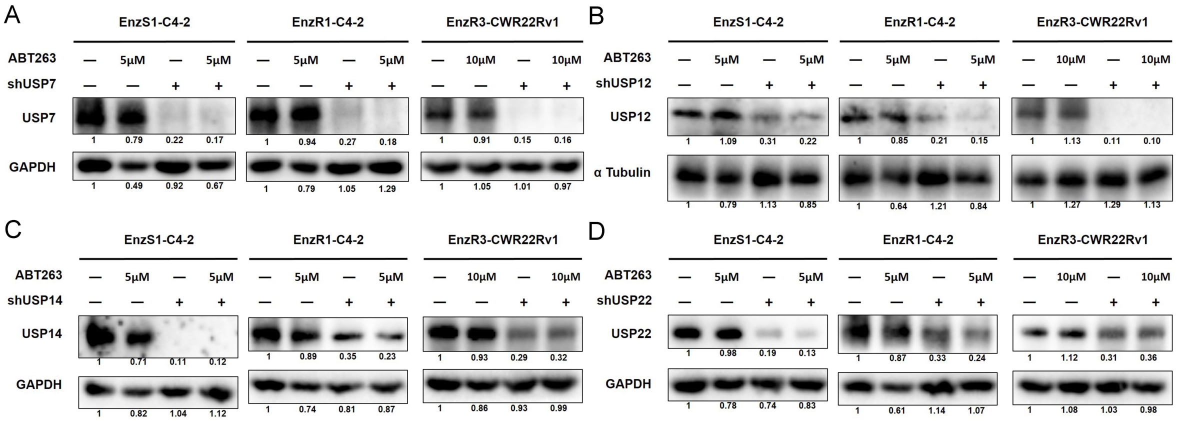

Supplement Fig. 6. Confirming the knock down efficiency of shUSPs. Western blot assays were applied to confirm the knock down efficiency of shUSP7 (**A**), shUSP12 (**B**), shUSP14 (**C**), and shUSP22 (**D**). Data are presented as Mean ± SD.

Supplement Fig. 7

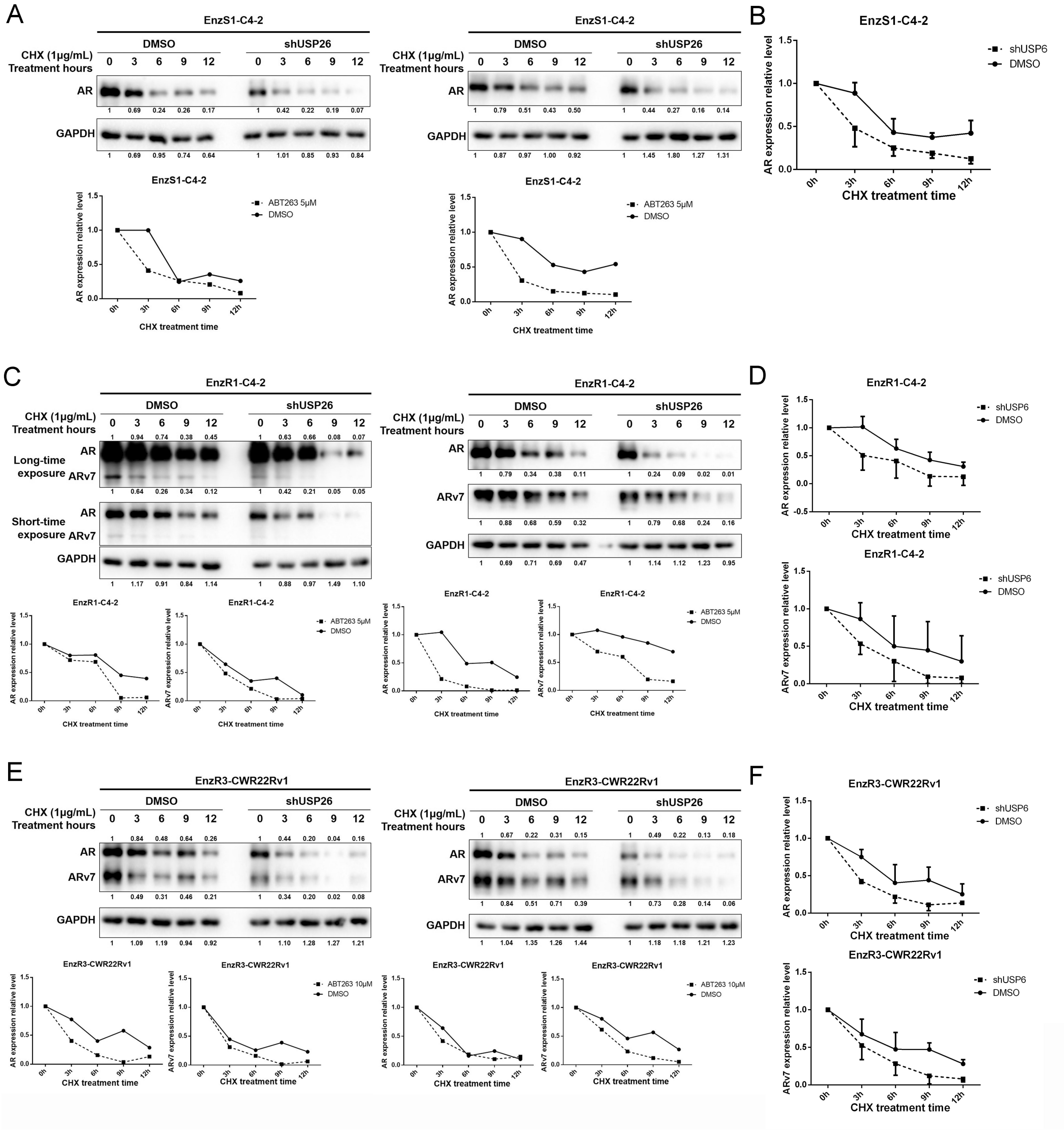

Supplement Fig. 7. USP26 contributes to protein stability of AR and ARv7. As described in **Fig. 6**, stability of AR and ARv7 in EnzS1-C4-2 cells (**A**), in EnzR1-C4-2 cells (**C**) and in EnzR3-CWR22Rv1 cells (**D**) was measured by western blot assay for another two times. Together with data in **Fig. 6A-C**, line graphs of AR/ARv7 protein stability were drawn, showing similar decreasing trend of AR/ARv7 stability in EnzS1-C4-2 (**B**), EnzR1-C4-2 (**D**), and EnzR3-CWR22Rv1 (**F**) cells after knocking down USP26. Data are presented as Mean ± SD.

Supplementary Table:

Table S1: Plasmid information used in the study

Gene_ID	Vector	Application	Sequence
USP7	pLKO.01	Knocking down	F: CCGGTAGTATCTTGAATAAATCCTGCTCTTTGGATCCGAGAGC<br>AGGATTTATTCAAGATACTATTTTTG<br>R: AATTCAAAAATAGTATCTTGAATAAATCCTGCTCTCGGATCCAA<br>AGAGCAGGATTTATTCAAGATACTA
USP12	pLKO.01	Knocking down	F: CCGGCGTAGAATTCTTCAATAGCTTGTGCTTGGATCCGGCACA<br>AGCTATTGAAGAATTCTACGTTTTTG<br>R: AATTCAAAAACGTAGAATTCTTCAATAGCTTGTGCCGGATCCAA<br>GCACAAGCTATTGAAGAATTCTACG
USP14	pLKO.01	Knocking down	F: CCGGACTGCTTGTAAGTCATAGTATCCACTTGGATCCGGTGGA<br>TACTATGACTTACAAGCAGTTTTTTG<br>R: AATTCAAAAAACTGCTTGTAAGTCATAGTATCCACCGGATCCAA<br>GTGGATACTATGACTTACAAGCAGT
USP22	pLKO.01	Knocking down	F: CCGGGGTACTGTCCATTCATCCTGCTCTCTTGGATCCGGAGAG<br>CAGGATGAATGGACAGTACCTTTTTG<br>R: AATTCAAAAAGGTACTGTCCATTCATCCTGCTCTCCGGATCCAA<br>GAGAGCAGGATGAATGGACAGTACC
USP26	pLKO.01	Knocking down	F: CCGGAATGTGTGGAGCCAGGATATCTTGGATCCGGATATCCTG<br>GCTCCACACATTTTTTTG<br>R: AATTCAAAAAAATGTGTGGAGCCAGGATATCCGGATCCAAGATA<br>TCCTGGCTCCACACATT
